# Maf1 Ameliorates Sepsis-Associated Encephalopathy by Suppressing the NF-*k*B/NLRP3 Inflammasome Signaling Pathway

**DOI:** 10.3389/fimmu.2020.594071

**Published:** 2020-12-23

**Authors:** Shenglong Chen, Chaogang Tang, Hongguang Ding, Zhonghua Wang, Xinqiang Liu, Yunfei Chai, Wenqiang Jiang, Yongli Han, Hongke Zeng

**Affiliations:** ^1^ Department of Critical Care Medicine, Guangdong Provincial People’s Hospital, Guangdong Academy of Medical Sciences, Guangzhou, China; ^2^ Department of Cerebrovascular Disease, The Fifth Affiliated Hospital of Sun Yat-Sen University, Zhuhai, China; ^3^ Department of Gerontological Critical Care Medicine, Guangdong Provincial People’s Hospital/Guangdong Academy of Medical Sciences/Guangdong Provincial Geriatrics Institute, Guangzhou, China; ^4^ Anesthesiology Department of Guangdong Cardiovascular Institute, Guangdong Provincial People’s Hospital, Guangdong Academy of Medical Sciences, Guangzhou, China

**Keywords:** sepsis-associated encephalopathy, Maf1, blood–brain barrier disruption, NF-*κ*B, NLRP3 inflammasome

## Abstract

**Background:**

The NOD-, LRR- and pyrin domain-containing protein 3 (NLRP3) inflammasome has been identified as an important mediator of blood–brain-barrier disruption in sepsis-associated encephalopathy (SAE). However, no information is available concerning the critical upstream regulators of SAE.

**Methods:**

Lipopolysaccharide (LPS) was used to establish an *in vitro* model of blood–brain barrier (BBB) disruption and an *in vivo* model of SAE. Disruption of BBB integrity was assessed by measuring the expression levels of tight-junction proteins. NLRP3 inflammasome activation, pro-inflammatory cytokines levels, and neuroapoptosis were measured using biochemical assays. Finally, the FITC-dextran Transwell assay and Evan’s blue dye assay were used to assess the effect of Maf1 on LPS-induced endothelial permeability *in vitro* and *in vivo.*

**Results:**

We found that Maf1 significantly suppressed the brain inflammatory response and neuroapoptosis induced by LPS *in vivo* and *in vitro*. Notably, Maf1 downregulated activation of the NF-*κ*B/p65-induced NLRP3 inflammasome and the expression of pro-inflammatory cytokines. In addition, we found that Maf1 and p65 directly bound to the *NLRP3* gene promoter region and competitively regulated the function of NLRP3 in inflammations. Moreover, overexpression of NLRP3 reversed the effects of p65 on BBB integrity, apoptosis, and inflammation in response to LPS. Our study revealed novel role for Maf1 in regulating NF-*κ*B-mediated inflammasome formation, which plays a prominent role in SAE.

**Conclusions:**

Regulation of Maf1 might be a therapeutic strategy for SAE and other neurodegenerative diseases associated with inflammation.

## Highlights

1. Maf1 alleviated the BBB disruption, neuro-apoptosis, and inflammation induced by LPS.
2. Maf1 suppressed inflammation in the LPS-induced *in vitro* BBB model by inactivating NLRP3 inflammasomes.
3. Overexpression of p65 reversed the effects of Maf1 on *in vitro* BBB integrity, cell viability, and apoptosis in response to LPS. Maf1 and p65 directly bound to the NLRP3 promoter.
4. Overexpression of NLRP3 reversed the effects of p65 on *in vitro* BBB integrity, apoptosis, and inflammation in response to LPS.
5. Maf1 improved cognitive impairment and *in vivo* BBB integrity.

## Introduction

Sepsis is s a life-threatening type of organ dysfunction caused by an infection (1, 2). Sepsis-associated encephalopathy (SAE) is a diffuse brain disorder caused by sepsis that occurs in the absence of a central nervous system (CNS) infection (2). The mortality rate of SAE patients can reach 70% (3), and the pathogenesis of SAE remains unclear. Previous reports have suggested that cerebrovascular dysfunction, oxidative stress, inflammatory damage, and mitochondrial dysfunction are related to the pathogenesis of SAE (4). However, the precise molecular mechanism of SAE has not been thoroughly investigated.

The blood–brain barrier (BBB) is a dynamic “physical barrier” that regulates the molecular flux between the blood and brain and thereby maintains a state of homeostasis in the CNS (5). The BBB is composed of endothelial cells, astrocytic end feet, and tight junctions (6, 7). The endothelial cells regulate BBB integrity, and due to their rapid growth and stability, are widely used to establish the *in vitro* BBB models (8). Recent studies have shown that a decrease in inter-endothelial junctions and an increase in endothelial barrier permeability are responsible for the pathogenesis of many cerebral diseases, such as SAE, Alzheimer’s disease, and cerebral ischemia (9).

BBB disruption is often associated with neuroinflammation (10). The excessive expression of pro-inflammatory cytokines (*e.g.*, IL-1β, IL-18, and TNF-α) often aggravates any existing damage in brain tissue (11). The initiation of SAE by treatment with lipopolysaccharides (LPSs) induces the translocation of NF-*κ*B into the nucleus, where it promotes the transcription of pro-inflammatory genes (12). NF-*κ*B activates a variety of genes in the nucleus, including microglial nod-like receptor protein 3 (*NLRP3*). NLRP3 plays an important role in the inflammation cascade by amplifying** an inflammation response (** 13 **)**; thus, inhibition of NLRP3 inflammasome activity has been proposed as a strategy for treating various diseases, including neurological diseases (14–17), osteoarthritis (18), diabetic nephropathy (19), liver fibrosis (20). Moreover, NF-*κ*B-induced NLRP3 expression is sufficient for NLRP3 to mediate inflammasome formation, suggesting that blocking the functional cross-talk that occurs between NF-*κ*B and NLRP3 might attenuate an inflammation-induced BBB injury.

Maf1 is a conserved negative regulator of RNA polymerase (pol) III (RNAP III) (21), which contains three conserved regions (A-. B-, and C-box). Maf1 participates in several physiological processes, including oncogenesis and lipid metabolism (22). Under nutrient-rich conditions, Maf1 is predominantly localized in the cytoplasm, whereas under* favorable growth conditions*, it binds to the RNAP III complex in the nucleus, resulting in the dissociation of RNAP III from tRNA promoter sequences (23). However, the role played by Maf1 and its underlying mechanisms in inflammation-induced BBB injuries remains unknown.

In this study, we used *in vitro *and* in vivo *BBB model to assess whether Maf1 could effect LPS-induced* SAE*. Furthermore, we explored how Maf1 regulated NF-*κ*B-induced NLRP3 activation. Our findings highlight novel of Maf1 in regulating NLRP3 activation and also suggest a potential therapeutic role for Maf1 in treating brain diseases associated with BBB disruption.

## Materials and Methods

### Cell Lines and Culture Conditions

BMECs and astrocytes were obtained from Cell Systems Corporation (ACBRI376, Kirkland, WA, USA) and cultured in DMEM containing 10% FBS in a 37°C incubator with a 5% CO_2_ atmosphere.

### Cell Treatments and Transfection

BMECs were incubated with or without LPS (1 μg/ml, Sigma-Aldrich, St. Louis, MO, USA) for 30 min. The cells were transfected with Maf1, p65, and NLRP3 overexpression plasmids or Maf1 and p65 knockdown plasmids for 48 h using Lipofectamine™ 2000 (Invitrogen, Carlsbad, CA, USA), and then incubated with LPS for 30 min. All plasmids were obtained from Genepharma (Shanghai, China).

### Establishment of the *In Vitro* BBB Model

An *in vitro* model of the BBB was constructed by co-culturing mouse brain microvascular endothelial cells (BMECs) and astrocytes, as previously described (24). Astrocytes (1.5 × 104) were seeded into the lower chambers of a 24-well Transwell plate and allowed to adhere overnight (considered as day −1). On the next day (day 0), BMECs (1.5 × 105), which had been transfected with the indicated plasmid (day −2) or treated with LPS for 48 h, were seeded into the upper chambers of the 24-well Transwell plate that had been coated with fibronectin. The BMECs were cultured in DMEM containing 10% FBS.

### Transendothelial Electrical Resistance

The barrier integrity of BMECs was measured in units of TEER using a1600R ECIS system at 0, 6, 12, and 24 h (day 0). The readings were acquired continuously at 4,000 Hz and at 30 min intervals.

### *In Vitro* Permeability Assessments

Cells from different groups were seeded into a Transwell chamber (Corning Costar, Corning, NY, USA) at a density of 1 × 104 cells/chamber and cultured for 72 h. After discarding the medium, the cells in the upper chamber were incubated with 10 kDa FITC-dextran (10 mg/ml; 10 μl) for 1 h; after which, the fluorescence intensity in the upper and lower chambers was determined with a fluorescence microplate reader (M200 PRO, Tecan, Switzerland). Based on the relative fluorescent units in the upper and lower chambers, the permeability coefficient was determined using a previously reported formula (25). All independent experiments were performed in triplicate.

### Cell Counting Kit-8 Assay

After being co-cultured for 3 days, BMECs were seeded into the wells of 96-well plates at a density of 3,500 cells per well and cultured for different time periods (0, 12, and 24 h). Next, 10 μl of CCK-8 reagent (Promega, Madison, WI, USA) was added to each well, and the cells were incubated for 2 h at 37°C. The optical density (OD) of each well was 450 nm, which was measured with a microplate reader.

### Flow Cytometry Analysis of Apoptosis

After being co-cultured for 3 days, BMECs were collected for an analysis of apoptosis that was performed using an Annexin V/PI Apoptosis Detection kit (Beyotime Institute of Biotechnology). In brief, the cells were re-suspended and then incubated with 10 μl of Annexin-V and 10 μl of PI for 15 min in the dark. Subsequently, the apoptotic rate was determined with a FACSAria flow cytometer (BD Biosciences, Franklin Lakes, NJ, USA).

### Dual-Luciferase Reporter Assay

The wild-type (WT) and mutated (MUT) putative Maf1-binding sites in the NLRP3-promoter region were cloned into the upstream region of the pGL3-REPORT luciferase vector (Promega). For the reporter assay, HEK293T cells were co-transfected with the above NLRP3 promoter-luciferase reporter constructs and pcDNA4.0/pcDNA4.0-Maf1 plasmids for 48 h by using Lipofectamine 2000 (Invitrogen). Next, relative luciferase activities were measured using a dual-Luciferase reporter assay system (Promega). Each transfection was performed in triplicate.

### Electrophoretic Mobility Shift Assay

The interaction between Maf1 and NLRP3 was examined using an EMSA Gel Shift Kit (Viagene, Tampa FL, USA) in accordance with the manufacturer’s instructions. Cell nuclear extracts were isolated and incubated with biotin-labeled probes containing the NLRP3 consensus sequence. Next, the specific anti-Maf1 or anti-p65 antibody was added to the mixture of nuclear extracts and DNA probes. The DNA–protein complexes were transferred onto a nylon binding membrane and detected using a streptavidin-horseradish peroxidase conjugate enhanced chemiluminescence (ECL) detection system (Sage Creation, Beijing, China).

### Chromatin Immunoprecipitation Assay

CHIP assays were performed using an Agarose CHIP Kit (Thermo Fisher, Waltham, MA, USA) according to the manufacturer’s instructions. Briefly, cells transfected with pcDNA4.0-Maf1 or pcDNA4.0-p65 plasmids were harvested, cross-linked by 1% formaldehyde for 10 min, and then treated with glycine for 5 min to stop the cross-linking. Nuclear lysates were sonicated for 20 min using a Scientz-IID sonicator (Scientz, Zhejiang, China). Immunoprecipitation was conducted with specific antibodies (anti-Maf1, anti-p65) or isotype IgG antibodies. Following proteinase K digestion, bound target DNA fractions were amplified for a region that spanned nucleotides containing the Maf1 or p65 binding sites of the *NLRP3* promoter.

### Sepsis Associated Encephalopathy Rat Model and Groups

Adult male Sprague–Dawley rats (n = 48; weight range 180–220 g) were purchased from the animal center of Sun Yat-Sen University (Guangzhou, China) and housed in a room maintained at 22–25°C and 40–50% humidity. Food and water were available *ad libitum*. The rats were randomly assigned to four different groups. Rats in the Sham group (n = 12) received equal volumes of saline; rats in the SEA group (n = 12) received intraperitoneal injections of LPS dissolved in saline (5 mg/kg weight); rats in the SAE + NC group (n = 12) received intraperitoneal injections of LPS (5 mg/kg body weight), and the control plasmid (100 μg) was injected *via* the carotid artery; rats in the SAE + Maf group (n = 12) were intraperitoneally injected with LPS (5 mg/kg weight), and theMaf1 overexpression plasmid (100 μg) was injected *via* the carotid artery. After undergoing surgery and treatments, the rats allowed to recover for 24 h and had free access to food and water. All animal experiments were conducted according guidelines in the *National Institutes of Health Guide for the Care and Use of Laboratory Animals*. This study protocol was approved by the Animal Care Committee of Guangdong Provincial People’s Hospital.

### Animal Sample Preparation

At the end of the Morris Water Maze (MWM) test, rats were anesthetized with 40% urethane and sacrificed. The brain of each rat was removed, and specimens of whole brain tissue (n = 6 for each group) were used for Evans blue assays. Samples of brain cortex tissue (n = 6 for each group) were collected for analysis by Western blotting, IHC, and ELISA assays.

### *In Vivo* BBB Permeability Assessments

Evan’s blue dye assays were performed to assess the *in vivo* permeabilization of the BBB in the SAE model rats, as previously described (26). In brief, six rats from each group were anesthetized with 40% urethane; after which, they were injected with 2% solution of Evans Blue in sterile saline solution *via* the tail vein, and then transcardially perfused with cold saline. Next, the brain tissues were quickly removed, weighted, and homogenized in 1.5 ml ice-cold PBS. After centrifugation at 6,000×g for 30 min, the absorbance of each supernatant at 620 nm was measured with a spectrophotometer.

### Morris Water Maze Test

The MWM test was performed to determine the learning and memory abilities of each rat as previously described (27). Briefly, the water maze consisted of a circular black pool measuring 100 cm in diameter and 38 cm in height. The pool was filled with opaque water (black ink) at a temperature of 23 ± 1°C, with a depth of 25 cm. A submerged (1.5 cm beneath the water surface) platform was located at a fixed point in the pool (the target quadrant). The movement tracks of each rat were video-recorded and automatically scored using Smart tracking software (ANY-maze; Stoelting, Wood Dale, IL, USA). The escape latency to reach the platform and the platform-crossing times were analyzed.

### Enzyme Linked Immunosorbent Assay

The supernatant of cultured cells were analyzed by ELISA. Samples of cerebral cortex tissue (n = 6 for each group) were homogenized in 0.2 ml of ice-cold PBS and centrifuged at 6,000×g for 30 min. The levels of IL-1β and IL-18 in each supernatant were analyzed using ELISA assay kits according to the instructions provided by the manufacturer. All samples were analyzed in duplicate.

### Immunofluorescence Assay

For immunocytochemistry, growth phase cells from different groups were plated onto coverslips and incubated overnight. After being rinsed twice with cold PBS, the cells were fixed in 4% paraformaldehyde for 30 min, permeabilized with 0.2% Triton X-100 and then incubated with primary antibodies against Maf1, p65, and NLRP3 at room temperature for 2 h. Immunoreactivity was visualized using AF-594 (red) or AF488 (green)-conjugated secondary antibodies and counterstained with 4′,6-diamidino-2-phenylindole (DAPI). The co-expression of Maf1 and NLRP3, as well as Maf1 and p65, was viewed using a Leica TCS SP5-X inverted confocal microscope (Leica Microsystems, Buffalo Grove, IL).

### Immunohistochemistry

Samples of brain tissue (n = 6 for each group) were fixed in 4% paraformaldehyde, embedded in paraffin, and cut into 4 μm thick sections. After dewaxing, dehydration, and antigen retrieval, the tissue specimens were blocked and incubated with a Bax or Bcl-2 antibody. The subsequent steps were the same as those performed for immunofluorescence assays.

### Western Blot Analysis

Cytosolic and nuclear fractions were prepared from the cell culture using Nuclear-Cytosol Extraction Kit (TDY, Biotech CO., Ltd, Beijing). The total proteins were extracted from brain tissues (cerebral cortex, n = 6 for each group) using RIPA lysis buffer (Beyotime Biotechnology, Shanghai, China), and the protein concentration of each extract was determined using a BCA protein assay (Pierce, Rockford, IL, USA). Next, an equal amount (30 μg) of protein from each extract was separated by 10–15% sodium dodecyl sulfate-polyacrylamide gel electrophoresis (SDS-PAGE), and the protein bands were transferred onto PVDF membranes, which were subsequently blocked with 5% skimmed milk for 2 h. The membranes were then incubated overnight at 4°C with primary antibodies against Maf1 (Abcam, Cambridge, UK, ab230499, 1:500 dilution), NF-*κ*B p65 (CST, Danvers, MA, USA, 8242, 1:1,000 dilution), NLRP3 (Abcam, ab214185, 1:500 dilution), Claudin-5 (Abcam, ab131259, 1:5,000 dilution), Occludin (Abcam, ab167161, 1:50,000 dilution), ZO-1 (Abcam, ab96587, 1:2,000 dilution), Caspase-1 (Abcam, ab1872, 1:500 dilution), Bax (Abcam, ab32503, 1:5,000 dilution), Bcl-2 (Abcam, ab196495, 1:1,000 dilution), IL-1β (Abcam, ab239517, 1:1,000 dilution), IL-18 (Abcam, ab191860, 1:1,000 dilution), GSDMD (Abcam, ab219800, 1:1,000 dilution), Histone 3 (Abcam, ab1791, 1:3,000 dilution), and GAPDH (Abcam, ab181602, 1:10,000 dilution), followed by incubation with horseradish peroxidase-conjugated antibodies for 2 h at room temperature. The immunoreactive protein bands were visualized with an enhanced chemiluminescence (ECL) kit (Shanghai Sangon, China). GAPDH and *β*-actin served as internal control for total protein and cytoplasm protein, respectively. Histone3 served as internal control for the nucleus.

### Statistical Analysis

All statistical analyses were performed using SPSS Statistics for Windows, Version 17.0 software (SPSS, Inc, Chicago, IL, USA). Results were expressed as mean value  ±  standard deviation (SD) of data obtained from at least three independent experiments. Comparisons of normally distributed data between two groups were performed using the Student’s t-test. Normally distributed data from multiple groups was compared using one-way analysis of variance (ANOVA) followed by Bonferroni’s *post hoc* test. Comparisons of non-normally distributed data between two groups were made using the Mann–Whitney U test, and the Kruskal–Wallis ranked analysis was used to compare non-normally distributed data between more than two groups. A *p*-value <0.05 was considered to be statistically significant.

## Results

### Maf1 Alleviates the BBB Disruption, Neuro-Apoptosis, and Inflammation Induced by LPS

**Figure 1:**
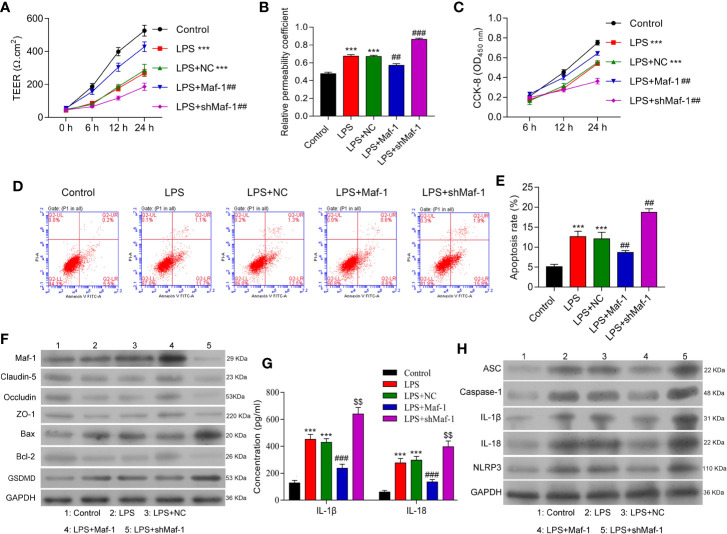
Maf1 alleviated BBB disruption, neuro-apoptosis, and inflammation induced by LPS. BMECs were transfected with a control vector (NC), Maf1 or shMaf1 plasmid, followed by LPS stimulation; they were then co-cultured with astrocytes to establish an *in vitro* BBB model. **(A)** The TEER values of monolayer cells at 0, 6, 12, and 24 h were measured using a bioelectric impedance analyzer. **(B)** Trans-endothelial permeability assays were performed to assess the *in vitro* BBB integrity by using the diffusion of 10 kDa FITC-dextran method. The relative permeability coefficient was determined after Transwell experiments were conducted. **(C)** Cell viability was analyzed using the CCK-8 assay. **(D)** Representative images of Annexin V/PI double staining, and **(E)** measurements of apoptosis. **(F)** The levels of Maf1, Claudin-5, Occludin, ZO-1, Bax, Bcl-2, and GSDMD were determined by western blotting. GAPDH served as a loading control. **(G)** Cell-free conditioned culture medium was collected and analyzed by ELISA for IL-1β and IL-18 levels. **(H)** Western blot analyses of pro-inflammatory cytokine (ASC, Caspas-1, IL-1β, andIL-18) and NLRP3expression levels. Results are expressed as the mean value ± SD of data obtained from three separate experiments. ***p< 0.001, compared with Control; ^##^ *p* < 0.01, compared with LPS + NC.

Disruption of the BBB and increased apoptosis have both been implicated in the pathogenesis of SAE (28, 29). We determined the effects of Maf1 on the permeability of BMECs after treatment with LPS. As shown in **Figure 1A**, the tight-junctions of endothelial cells were disrupted by LPS stimulation. There was evidence of endothelium detachment, and areas of reduced electron density were seen (*p* < 0.001). However, these effects were reversed to some extent by Maf1 overexpression and further aggravated by Maf1 knockdown (*p* < 0.01). In addition, the permeability of the cell monolayer formed across the Transwell chamber after LPS stimulation was partially attenuated by Maf1 overexpression, but aggravated by Maf1 knockdown (**Figure 1B**, *p* < 0.01 and *p* < 0.001). We also observed that the viability of BMECs was significantly decreased by LPS stimulation (*p* < 0.001), but that decrease could be recovered by Maf1 overexpression (*p* < 0.01) (**Figure 1C**). Moreover, the cell apoptosis induced by LPS was reversed by Maf1 and enhanced by inhibition of Maf1 (**Figures 1D, E**, *p* < 0.01). We also detected the expression of apoptotic- and tight junction-related proteins in the *in vitro* BBB model, and found that the levels of tight junction-related proteins (Occludin, Claudin-5, and ZO-1) were decreased, the levels of the pro-apoptotic protein Bax and pyroptosis protein GSDMD were increased, while the levels of the anti-apoptotic protein Bcl-2 were decreased after LPS stimulation. Notably, the effects of LPS on expression of the above proteins were reversed by Maf1 overexpression but enhanced by Maf1 knockdown (**Figure 1F** and **Supplementary Figure 1A**).

Neuroinflammation is a common feature of SAE and is partly driven by inflammasome formation and the subsequent release of other pro-inflammatory factors (30, 31). We found that the levels of IL-1β and IL-18 in co-cultured BMECs were increased by LPS stimulation and decreased by Maf1 overexpression, and Maf1 knockdown further increased the levels of IL-1β and IL-18 induced by LPS (**Figures 1G, H** and **Supplementary Figure 1B**, *p* < 0.001). Moreover, our Western blot studies showed that the degree of NLRP3 inflammasome activation induced by LPS could be attenuated by transfection with Maf1, but was further enhanced by Maf1 knockdown (**Figures 1G, H** and **Supplementary Figure 1B**, *p* < 0.001). These data showed that Maf1 modulated inflammation in BMECs.

### Maf1 Suppressed Inflammation in the LPS-Induced *In Vitro* BBB Model by Inactivating NLRP3 Inflammasomes

**Figure 2:**
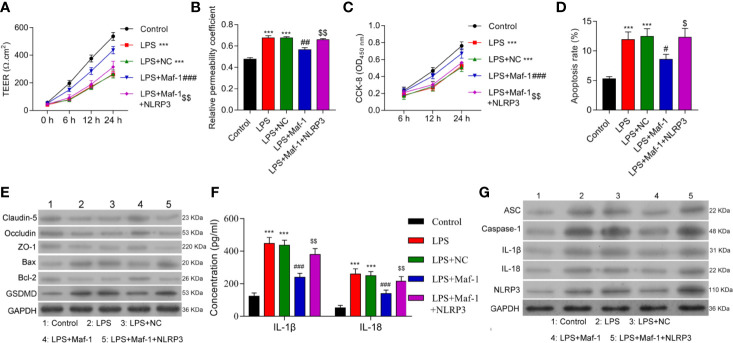
Maf1 suppressed inflammation in the LPS-induced *in vitro* BBB model through inactivation of the NLRP3 inflammasome. BMECs were transfected with the control vector (NC), Maf1 or a combination of Maf1and NLRP3, followed by LPS stimulation. The BMECs were then co-cultured with astrocytes to establish an *in vitro* BBB model. **(A)** The TEER values of monolayer cells at 0, 6, 12, and 24 h were measured using a bioelectric impedance analyzer. **(B)** Trans-endothelial permeability assays were performed to assess *in vitro* BBB integrity by using the diffusion of 10 kDa FITC-dextran method. The relative permeability coefficient was determined after the Transwell experiment. **(C)** Cell viability was analyzed using the CCK-8 assay. **(D)** Quantification of cell apoptosis. **(E)** Western blot results for the levels of Claudin-5, Occludin, ZO-1, Bax, Bcl-2, and GSDMD. **(F)** The cell-free conditioned culture medium was collected and analyzed IL-1β and IL-18 levels *via* ELISA **(G)** Western blot analyses for pro-inflammatory cytokine (ASC, Caspase-1, IL-1β, and IL-18) and NLRP3 expression levels. Results are expressed as the mean value ± SD of data obtained from three separate experiments. ***p < 0.001, compared with Control; ^#^ *p* < 0.05, ^##^ *p* < 0.01 and ^###^ *p* < 0.001, compared with LPS + NC; ^$^ *p* < 0.05 and ^$$^ *p* < 0.01 compared with LPS + Maf-1.

To further understand the role played by Maf1 in regulating the BBB, we investigated whether the modulation of BBB integrity, apoptosis, and inflammation by Maf1 depended on the activation of NLRP3. We found that overexpression of NLRP3 reversed the protective effect of Maf1 on BBB disruption (**Figures 2A, B**, *p* < 0.01). The ability of Maf1 to regulate cell proliferation, apoptosis, and inflammation was repressed by NLRP3 (**Figures 2C–E** and **Supplementary Figure 2A**, *p* < 0.05 and *p* < 0.01**)**. Notably, the levels of tight junction-related proteins (Occludin, Claudin-5, and ZO-1) were decreased, the levels of pro-apoptotic protein (Bax) and pyroptosis protein GSDMD were increased, the levels of anti-apoptotic protein Bcl-2 were decreased, and the levels of inflammatory cytokines, IL-1β and IL-18 were increased in LPS-treated cells after co-expression of Maf1 and NLRP3 (**Figures 2F, G** and **Supplementary Figure 2B**, *p* < 0.01). These results showed that Maf1 protects against BBB disruption by inactivating NLRP3.

### Overexpression of p65 Reversed the Effects of Maf1 on *In Vitro* BBB Integrity, Cell Viability, and Apoptosis in Response to LPS

**Figure 3:**
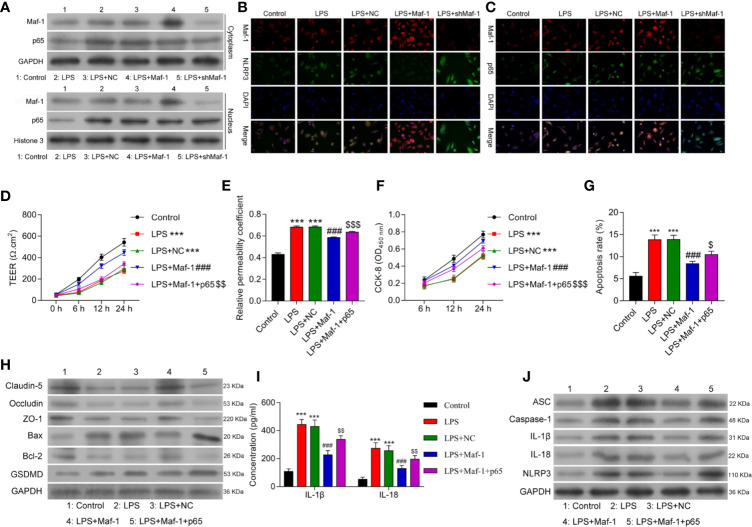
Overexpression of p65 reversed the effects of Maf1 on *in vitro* BBB integrity, cell viability, and apoptosis in response to LPS. Cells were treated as shown in **Figure 1**. **(A)** The cytoplasm and nucleus were separated. Western blot analyses of Maf1 and p65 expression in the cytoplasm and nucleus. **(B)** The co-location and expression levels of Maf1 and NLRP3 were determined by immunofluorescence assays. **(C)** Immunofluorescence staining of Maf1 and p65 is shown. BMECs were transfected with the control vector (NC), Maf1, or a combination of Maf1and p65, followed by LPS stimulation. The BMECs were then co-cultured with astrocytes to establish an *in vitro* BBB model. **(D)** The TEER of values of monolayer cells at 0, 6, 12, and 24 h were measured using a bioelectric impedance analyzer. **(E)** Trans-endothelial permeability assays were performed to assess *in vitro* BBB integrity by using the diffusion of 10 kDa FITC-dextran method. The relative permeability coefficient was determined after the Transwell experiment. **(F)** Cell viability was analyzed using the CCK-8 assay. **(G)** Quantification of cell apoptosis. **(H)** Western blot results for the levels of Claudin-5, Occludin, ZO-1, Bax, Bcl-2, and GSDMD. **(I)** The cell-free conditioned culture medium was collected and analyzed for IL-1β and IL-18 levels *via* ELISA **(J)** Western blot analyses of pro-inflammatory cytokine (ASC, Caspase-1, IL-1β andIL-18) and NLRP3 expression levels. Data are expressed as the mean value ± SD of data obtained from three separate experiments. ***p < 0.001, compared with Control; ^###^ *p* < 0.001, compared with LPS + NC; ^$^ *p* < 0.05, ^$$^ *p* < 0.01 and ^$$$^ *p* < 0.05 compared with LPS+Maf-1.

Inflammasome formation depends on the interaction of the NF-*κ*B p65 protein with NLRP3 under conditions of stress, which includes BBB disruption. Based on this, we next examined how Maf1 might play a role in regulating NF-*κ*B-induced NLRP3 inflammasome formation. As shown in **Figure 3A**, the levels of Maf1 and p65 expression in the cytoplasm and nucleus were up-regulated after LPS stimulation. Maf1 overexpression promoted the entry of Maf1 into the nucleus, while Maf1 silencing had the opposite effect. Immunofluorescence assays were conducted to confirm the expression and location of Maf1, p65, and NLRP3. As depicted in **Figure 3B**, Maf1 and p65 were co-located in the nucleus, and the level of p65 expression did not obviously change under conditions of either Maf1 overexpression or knockdown. Maf1 and NLRP3 were also co-localized in the nucleus, and NLRP3 expression was significantly downregulated when Maf1 was overexpressed (**Figure 3C**).

Next, we overexpressed both Maf1 and p65 in the *in vitro* BBB model (**Supplementary Figure 3A**). As shown in **Figure 3D**, the numbers of endothelial cell tight junctions disrupted by LPS stimulation were significantly reduced when Maf1 overexpressed (*p* < 0.001). However, overexpression of p65 reversed this protective effect of Maf1 (*p* < 0.01). Similarly, the effects of Maf1 overexpression on monolayer permeability, cell viability, and apoptosis after LPS stimulation were repressed by p65 overexpression (**Figures 3E–H** and **Supplementary Figure 3B**). We also examined the numbers of tight junctions and expression of inflammatory cytokines, and found that p65 overexpression attenuated the protective effects of Maf1 **(** **Figures 3H–J** and **Supplementary Figure 3C** **)**. More importantly, we found that NLRP3 expression was regulated by Maf1 and p65 (**Figure 3J**). These results suggest that Maf1 and p65 co-regulate NLRP3 inflammasome activation.

### Maf1 and p65 Directly Binding to the NLRP3 Promoter

**Figure 4:**
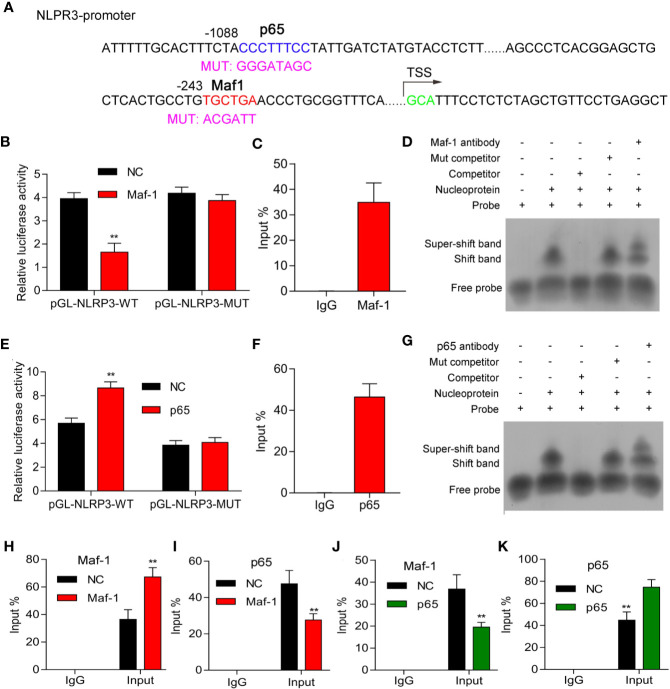
Maf1 and p65 directly bound to the NLRP3 promoter. **(A)** The mutant binding site of the *NLPR3*promoter. **(B, E)** HEK293T cells were co-transfected with pcDNA4.0 or pcDNA4.0-Maf1/pcDNA4.0-p65 vectors and pGL-NLRP3-WT orpGL-NLRP3-MUT vectors, and the relative luciferase activities are shown. **(C, F)** ChIP assays were performed to determine the binding of Maf1 and p65 to the *NLRP3* promoter in BMECs *in vitro*. **(D, G)** EMSA assays were performed to detect the direct binding of Maf1 and p65 to the *NLRP3* promoter in BMECs *in vitro*. **(H–K)** ChIP assays were performed to explore howMaf-1 or p65 regulated the *NLRP3* promoter in BMECs. Total chromatin from cell culture as the input, and normal rabbit IgG served as the negative control antibody. Results are expressed as the mean value ± SD of data obtained from three separate experiments, **p < 0.01, compared with NC.

To further explore the mechanism by which Maf1 and p65 regulate NLRP3, we performed luciferase reporter, ChIP, and EMSA assays to determine whether Maf1 could directly bind to the putative promoter region of NLRP3. The mutant binding site of the *NLPR3* promoter is shown in **Figure 4A**. We found that relative luciferase activity was significantly decreased when using the wild-type *NLRP3* promoter, while no obvious difference was found when using the mutant *NLRP3* promoter (**Figure 4B**, *p* < 0.01). Furthermore, results of ChIP and EMSA assays showed that Maf1 was directly bound to *NLRP3* promoter (**Figures 4C, D**). Moreover, we also found that p65 could directly bind to the putative binding region of the *NLRP3* promoter (**Figures 4E–G**).

Our data showed that the interaction between Maf1 and NLRP3 was enhanced by Maf1 overexpression (**Figure 4H**, *p* < 0.01), whereas the interaction between p65 and NLRP3 was attenuated by Maf1 overexpression (**Figure 4I**, *p* < 0.01). Conversely, the interaction between Maf1 and NLRP3 in BMECs was impaired, while the interaction between p65 and NLRP3 was strengthened under conditions of p65 overexpression (**Figures 4J, K**, *p* < 0.01**)**. These data indicate that Maf1 and p65 play opposite roles in regulating NLRP3 expression by competitively binding to the *NLRP3* promoter region in the *in vivo* model of LPS-induced BBB disruption.

### Overexpression of NLRP3 Reverses the Effects of p65 on *In Vitro* BBB Integrity, Apoptosis and Inflammasome in Response to LPS

**Figure 5:**
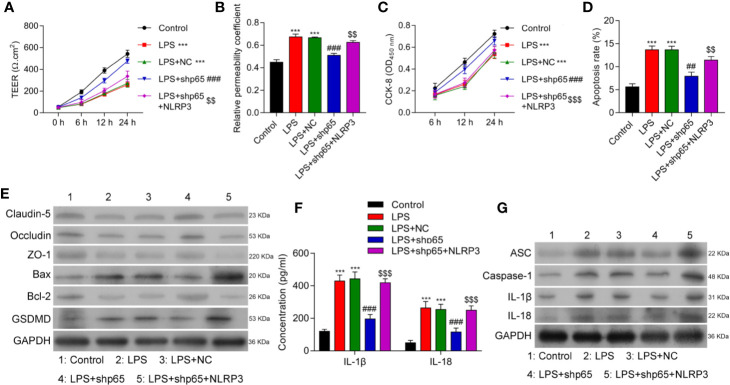
Overexpression of NLRP3 reversed the effects of p65 on *in vitro* BBB integrity, apoptosis, and inflammation in response to LPS. BMECs were transfected with the control vector (NC), NLRP3 or a combination of shp65 and NLRP3, followed by LPS stimulation. The BMECs were then co-cultured with astrocytes to establish an *in vitro* BBB model. **(A)** The TEER values of monolayer cells at 0, 6, 12, and 24 h were measured using a bioelectric impedance analyzer. **(B)** Trans-endothelial permeability assays were performed to assess *in vitro* BBB integrity by using the diffusion of 10 kDa FITC-dextran method. The relative permeability coefficient was determined after the Transwell experiment. **(C)** Cell viability was analyzed using the CCK-8 assay. **(D)** Quantification of cell apoptosis. **(E)** Western blot results for the levels of Claudin-5, Occludin, ZO-1, Bax, Bcl-2, and GSDMD. **(F)** The cell-free conditioned culture medium was collected and analyzed for IL-1β and IL-18 levels *via* ELISA. **(G)** Western blot analysis of pro-inflammatory cytokine (ASC, caspase-1, IL-1β, andIL-18) levels. Results are expressed as the mean value ± SD of data obtained from three separate experiments. ***p < 0.001, compared with Control; ^###^ *p* < 0.001, compared with LPS+NC; ^$$^ *p* < 0.01 and ^$$$^ *p* < 0.001 compared with LPS + shp65.

NLRP3 inflammasome activation is a two-step process involving the presence of inflammatory factors (*e.g.*, LPS) as a first signal to activate NF-*κ*B; this is followed by an increase in NLRP3 expression (19, 32). To clarify the role of NF-*κ*B in NLRP3 expression in LPS-induced inflammation, we inhibited p65 expression in cells treated with LPS. The levels of p65 protein were significantly decreased in cells that have been transfected with shp65 plasmids (**Supplementary Figure 4A**). As shown in **Figures 5A, B**, inhibition of p65 significantly reduced BBB disruption, while overexpression of NLRP3 reversed that effect (*p* < 0.001). Additionally, we found that cell proliferation, apoptosis, and tight junction formation were also regulated by NLRP3 (**Figures 5C–E** and **Supplementary Figure 4B**), and inflammatory cytokine levels were modulated by NLRP3 and p65 (**Figures 5F, G** and **Supplementary Figure 4C**). Our data showed that p65 NF-*κ*B induced NLRP3 activation by interacting with NLRP3.

### Maf1 Improved Cognitive Impairment and *In Vivo* BBB Integrity

**Figure 6:**
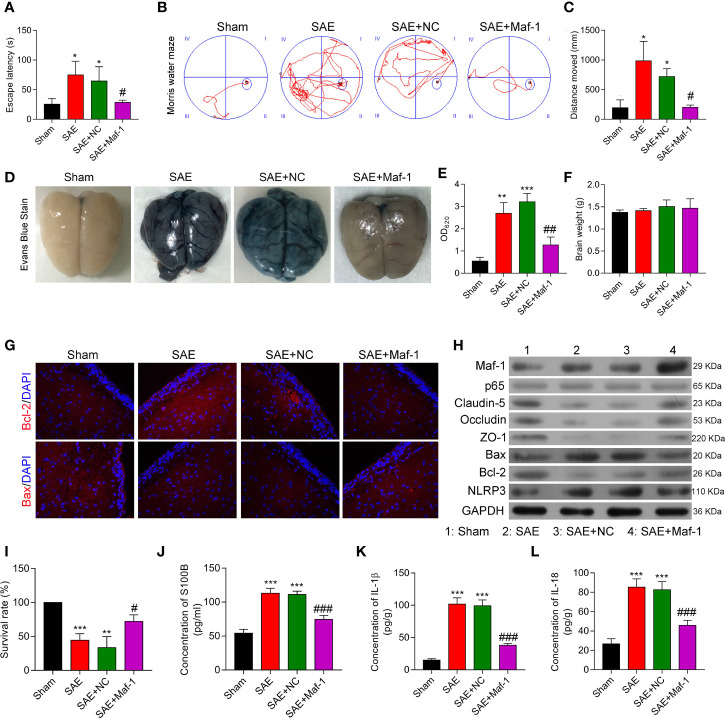
Maf1 improved cognitive impairment and *in vivo* BBB integrity. **(A)** Analysis of the escape latency time to the platform in different groups. **(B)** The swim paths of mice in the MWM test. **(C)** The distance of crossings over the platform location in the probe trial. **(D)** Evan’s blue dye in the brain tissues. **(E)** The concentrations of Evan’s blue were measured. **(F)** The weights of the brains. **(G)** Immunofluorescence staining of Bax and Bcl-2 in brain tissues. **(H)** Western blot results for levels of Maf1, p65, Claudin-5, Occludin, ZO-1, Bax, Bcl-2, and NLRP3. **(I)** The survival rate of rats in the different groups was calculated. **(J–L)** Cerebral cortex tissue was collected and analyzed for S100B **(J)**, IL-1β **(K)**, and IL-18 **(L)** *via* ELISA. Results are expressed as the mean value ± SD of data obtained from three separate experiments. *p < 0.05, **p < 0.01 and ***p < 0.001, compared with Sham; #, *p* < 0.05, ^##^ *p* < 0.01 and ^###^ *p* < 0.001, compared with SAE + NC.

To investigate whether Maf1 could exert its protective effects *in vivo*, we constructed a SAE rat model. Our data showed that the escape latency time was significantly prolonged in the SAE group, but the prolongation was reduced by carotid injection of Maf1 (**Figure 6A**, *p < 0.05*). MWM tests showed that rats in the SAE group traveled longer search routes than rats in the Sham group. However, rats in the SAE + Maf1 group showed a tendency to seek the platform in a direct manner when compared to rats in the SAE group (**Figure 6B**). Carotid injection of Maf1 also markedly decreased the distance that rats had to travel when comparing to rats in the SAE group (**Figure 6C**, *p* < 0.05). In addition to behavioral changes, BBB permeability, as indicated by higher levels of Evan’s blue dye in the brain tissues or absorption values at OD620 was increased in the SAE group, but was weakened following carotid injection of Maf1 (**Figures 6D, E**). The brain weights in all of the treatment groups remained unchanged (**Figure 6F**). The expression of pro-apoptotic Bax was up-regulated, and expression of anti-apoptotic Bcl-2 was down-regulated in the SAE group when compared with the Sham group (**Figures 6G, H**). However, those effects were obviously abolished by Maf1 (**Figures 6G, H** **).** At the end of the MWM test, we observed that the survival rate of rats with SAE was decreased. However, the survival rate could be increased by delivering Maf1 *via* carotid injection (**Figure 6I**). Moreover, the increased permeability of the BBB was associated with higher serum and cortex levels of S100B leakage in the SAE group, while Maf1 significantly decreased S100B expression (**Figure 6J**).

Consistent with the *in vitro* results, Maf1 significantly attenuated the increases in IL-1β and IL-18 in the SAE group and enhanced tight junction protein expression (**Figures 6H, K**, **L**). These *in vivo* results suggest that Maf1 plays a protective role in SAE by regulating inflammation and cell apoptosis.

## Discussion

In this study, we demonstrated that overexpression of Maf1 attenuated LPS-induced BBB disruption, and also reversed the downregulation of tight junction proteins. More importantly, Maf1 overexpression suppressed NLRP3 inflammasome formation and the release of pro-inflammatory proteins by directly binding the *NLRP3* promoter. Furthermore, we revealed that Maf1 competitively inhibited the binding of NF-*κ*B/p65 to the *NLRP3* promoter, and that Maf1 protects against BBB disruption induced by LPS *in vivo*.

Maf1 can function as global repressor of Pol III-dependent transcription in response to cellular stress (33). Hyperactive Pol III plays a role in human diseases as a master inhibitor of Pol III (33). Additionally, Maf1 is highly expressed in mouse brain, where it interacts with GABAARs (34). Injection of adenoviral-Maf1 into mice was shown to significantly alleviate aortic banding-induced cardiac hypertrophy by allowing direct binding of Maf1 to ERK1/2 (35). However, the effects of Maf1 on SAE have not been reported. Here, we showed that overexpression of Maf1 impaired LPS-induced BBB disruption and inflammation by inactivating NLRP3 inflammasomes. Moreover, Maf1 attenuated the increases in cell apoptosis and tight junction disruption induced by LPS. Our current data suggest that Maf1 helps to protect against BBB disruption.

BBB is a key determinant of SAE development, and functions to protect the CNS from pathogens and toxic stress (36). BBB function depends on the integrity of microvascular endothelial cells, which are connected by tight junctions (TJs) and adherent junctions (AJs) (37). Tight junction proteins such as Claudin-5, Occludin, ZO-1 in the brain endothelium are important for maintaining BBB integrity (38). Our data showed that the levels of tight junction proteins were decreased in the BBB cell model, while overexpression of Maf1 restored those protein levels. In addition, endothelial cell apoptosis is a secondary consequence of BBB disruption, along with the induced expression of apoptotic-related proteins (39). We revealed that the increased cell apoptosis and the decreased cell proliferation induced by LPS could be restored to some extent by Maf1 overexpression, and became further aggravated after Maf1 knockdown, suggesting that Maf1 helps protect against BBB disruption partly by regulating the expression of tight junction proteins and cell apoptosis.

Inflammatory responses, and especially inflammasome activation, are critical for the development of SAE (40–42). Among the many types of inflammasomes, the NLRP3 inflammasome is the most well characterized inflammasome and consists of NLRP3, ASC and Caspase-1 (43). Once activated, ASC self assembles (44) and activates pro-Caspase-1 (45). The activated caspase-1 then induces the maturation of IL-1β and IL-18 for subsequent releases (46). A previous study showed that the NLRP3 inflammasome plays a key role in causing endothelial cell injury and BBB disruption by modulating the HMGB1 pathway (47). When using our *in vitro* BBB model, we found that ASC, Caspase-1, and NLRP3 expression were all induced by LPS. Furthermore, the levels of both IL-1β and IL-18 were increased by LPS stimulation. However, overexpression of Maf1 partially reversed those effects, indicating that Maf1 helps to protect against BBB disruption by inactivating NLRP3 inflammasomes. More importantly, our data showed that overexpression of NLRP3 reversed the protective effects provided by Maf1 overexpression. Similarly, the modulatory effects of Maf1 on monolayer permeability, cell proliferation, cell apoptosis, and the expression of tight junction proteins after LPS stimulation were all reduced by overexpression of NLRP3. These results further confirmed that Maf1 protects against LPS-induced BBB disruption by inactivating NLRP3 inflammasomes. Previous studies revealed that NLRP3 deficiency decreases cerebral injury by reducing infarcts and BBB damage (48). A recent study showed that inhibition of NLRP3 by MCC950 alleviated the damage to the BBB after transient middle cerebral artery occlusion (tMCAO) (49). Moreover, *in vivo* studies have shown that a variety of pharmacological agents can ameliorate BBB disruption by suppressing activation of the NLRP3 inflammasome (50–53). Those reports and our results suggest that inactivation of the NLRP3 inflammasome helps to reduce BBB disruption.

Inflammasomes induce apoptosis and pyroptosis *via* complex molecular mechanisms. Inflammation activates caspase-1 and caspase-8, which induce cell death *via* multiple signal transducers, including GSDMD, Bid, and Caspase-7 (54). NLRP3-mediated IL-1β production requires NF-*κ*B activation and pro-inflammatory cytokines, suggesting that apoptosis drives NLRP3 inflammasome activation under inflammatory conditions (55). In addition, the NLRP3 inflammasome has been found to promote the maturation and secretion of pro-inflammatory cytokines and initiate pyroptosis (56). Moreover, Caspase-1 has the potential to induce pyroptosis and apoptosis, depending on expression of the pyroptosis mediator, gasdermin D (54). Here, we found that Maf1 not only reduced the expression of apoptotic proteins, but also GSDMD, which is a pyroptosis-related protein. Additionally, overexpression of NLRP3 reversed the protective effects of Maf1 on cell apoptosis and pyroptosis. Our data are the first to show that Maf1 helps to protect against BBB disruption by inhibiting NLRP3 inflammasome-induced cell apoptosis and pyrotosis.

The activation of the NLRP3 inflammasome is tightly regulated at the transcriptional and post-translational levels (57). The NLRP3 inflammasome can be activated by a variety of stimuli and multiple molecular and cellular events, including exposure to LPS (58). Several studies have indicated that both MAPK and NF-*κ*B play important roles in cellular responses to inflammation-induced stress and NLRP3 activation (57, 59). Many microbial components or cytokines can activate NF-*κ*B, which subsequently up-regulates NLRP3 and IL-1β (58). In gram-negative sepsis, LPS promotes the activation of NF-*κ*B, which leads to an induction of inflammatory mediators (60). P65, a subunit of the NF-*κ*B heterodimer, translocates into the nucleus where it induces the transcription of *NLRP3* and *IL-1β* (61). NLRP3 becomes activated by binding NF-*κ*B *via* a 1.3-kbp fragment located in the upstream transcriptional start site of the human NLRP3 gene (62). We found that Maf1 and p65 can directly bind to the promoter region of *NLRP3*. From a functional standpoint, knockdown of p65 repressed *in vitro* BBB integrity, cell apoptosis, and activation of the NLRP3 inflammasome in response to LPS stimulation; however, overexpression of NLRP3 repressed the protective effects provided by p65 knockdown. Our data suggest that p65/NF-*κ*B induces NLRP3 activation after LPS stimulation by interacting with NLRP3. In addition, a previous study showed that an NF-*κ*B inhibitor could significantly reduce activation of the NLRP3 inflammasome activation (63). In neurons and brain tissue exposed to *in vitro* and *in vivo* ischemic conditions, the NLRP3 inflammasome is activated by the NF-*κ*B and MAPK signaling pathways, while inhibitors of NF-*κ*B and MAPK reverse those effects (64). We found that knockdown p65 significantly inhibited activation of the NLRP3 inflammasome and also the degree of inflammation caused by LPS. Our data, as well as data gathered by other investigators, indicate that the NLRP3 inflammsome is modulated by the NF-*κ*B/p65 pathway, and suppression of the NLRP3 inflammasome helps to protect against BBB disruption.

Maf1 associates with RNA polymerase III and inhibits the recruitment of RNA polymerase III to the promoter region of its target DNA (65, 66). Under stress condition, Maf1 becomes dephosphorylated and migrates into the nucleus, where it decreases gene transcription (67). Here, we showed that Maf1 and p65 expression in both the cytoplasm and nucleus were up-regulated after LPS stimulation. Although Maf1 and p65 were co-located in the nucleus, p65 expression did not obviously change when Maf1 expression was up-regulated. Interestingly, the protective effects against *in vitro* BBB inflammation that were induced by Maf1 overexpression were restored by p65 overexpression, suggesting that Maf1 modulates the NLRP3 inflammasome *via* the p65 pathway. Moreover, both Maf1 and p65 were found to directly bind to the *NLRPS* promoter region and regulate NLRP3 expression, we showed that Maf1 and p65 played opposite roles in regulating NLRP3 expression by competitively binding to the *NLRP3* promoter, suggesting that Maf1 might act as a transcriptional regulator of NF-*κ*B/p65/NLRP3 to prevent BBB disruption in SAE. Our data suggest that Maf1 decreases NLRP3 expression by translocating into the nucleus, becoming co-localized with p65, and subsequently downregulating NLRP3 expression. Thus, we describe a new mechanism that regulates NLRP3 during LPS-induced BBB disruption.

**Figure 7:**
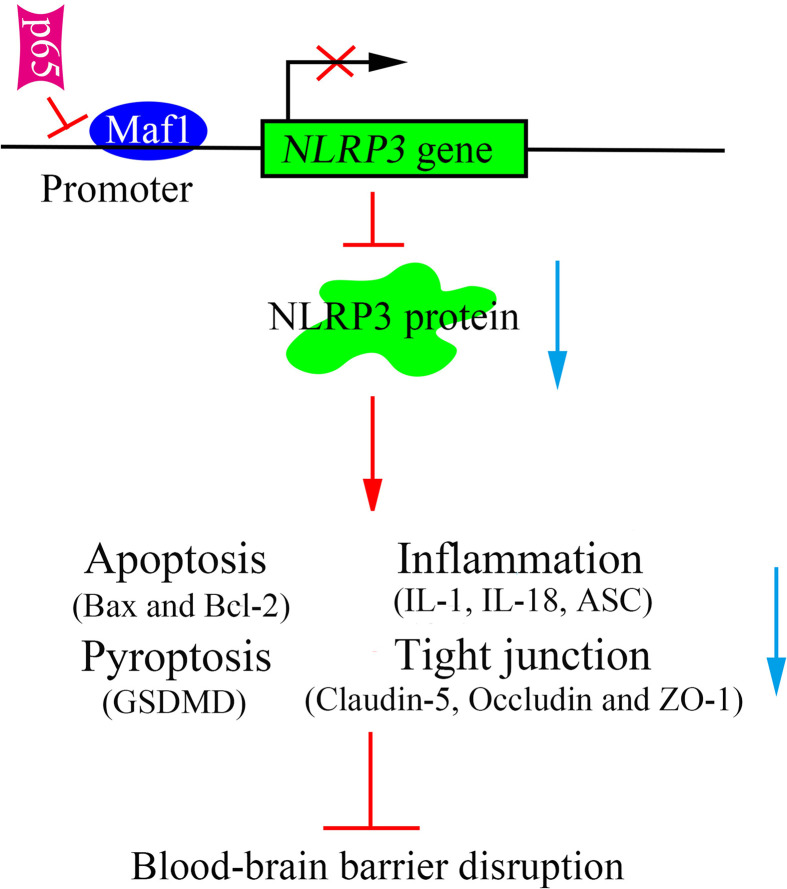
Schematic diagram of the proposed molecular mechanisms. A schematic diagram depicting the molecular mechanism by which Maf1 inhibits blood–brain barrier disruption by competing with p65 to regulate NLRP3 expression. Maf1 protects the blood–brain barrier by directly targeting NLRP3 to decrease apoptosis, the secretion of pro-inflammatory cytokines, blood–brain barrier permeability, and nerve injury.

In conclusion, this study revealed that Maf1 protects against LPS-induced BBB disruption in both *in vitro* and *in vivo*. Mechanistically, Maf1 suppressed the NF-*κ*B/NLRP3 inflammatory pathway by directly binding to the promoter region of *NLRP3*, thus reducing cell apoptosis and inflammation, and helping to protect against BBB disruption (**Figure 7**). Our findings reveal a new regulatory role for NLRP3 in LPS-induced BBB disruption, as it directly binds to the *NLRP3* promoter and its upstream regulator, NF-*κ*B/p65. This suggests that Maf1 helps to protect against SAE by inactivating NLRP3.

## Data Availability Statement

The original contributions presented in the study are included in the article/**Supplementary Materials**. Further inquiries can be directed to the corresponding author.

## Ethics Statement

The animal study was reviewed and approved by the Animal Care Committee of Guangdong Provincial People's Hospital.

## Author Contributions

SC, CT, and HD contributed equally to this work. SC, CT, and HZ conceived the study designed and planned. HD and ZW performed the assays in this research. HD and XL analyzed data and statistics. HD and YC interpreted data. SC and WJ prepared the manuscript. YH analyzed and searched literature. HZ collected the funds. All authors contributed to the article and approved the submitted version.

## Funding

This study was supported by the National Natural Science Foundation of China (81701875), Science and Technology Program of Guangzhou (201904010039, 202002030338), and The Science and Technology Project of Guangdong Province (2017A020215053), and Medical Scientific Research Foundation of Guangdong Province (A2019135).

## Conflict of Interest

The authors declare that the research was conducted in the absence of any commercial or financial relationships that could be construed as a potential conflict of interest.

## Supplementary Material

Supplementary Figure 1, related to Figure 1
**(B, C)**. A summary of western blot results from three independent experiments. **, *p* < 0.01 and ***, *p* < 0.001, compared with Control; #, *p* < 0.05, ##, *p* < 0.01 and ###, *p* < 0.001, compared with LPS + NC.

Supplementary Figure 2, related to Figure 2
**(B, C)**. A summary of western blot results from three independent experiments. *, *p* < 0.01 and ***, *p* < 0.001, compared with Control; #, *p*<0.05 and ##, *p*<0.01, compared with LPS + NC; $$, *p* < 0.01 and $$$, *p* < 0.001, compared with LPS+Maf-1.

Supplementary Figure 3, related to Figure 3
**(A)**. Expression of p65 after forcing overexpressionof p65 in BMSCs. **(B, C)**. A summary of western blot results from three independent experiments. ***, *p* < 0.001, compared with Control; #, *p* < 0.05 and ##, *p* < 0.01, compared with LPS+NC; $, *p* < 0.05 and $$, *p* < 0.01, compared with LPS+Maf-1.

Supplementary Figure 4, related to Figure 5
**(A)**. Expression of p65 after suppressing p65 expression in BMSCs. **(B, C)**. A summary of western blot results from three independent experiments. **, *p* < 0.01 and ***, *p* < 0.001, compared with Control; #, *p* < 0.05 and ##, *p* < 0.01, compared with LPS + NC; $$, *p* < 0.01 and $$$, *p* < 0.001, compared with LPS + shp65.

## Abbreviations

SAE, Sepsis-associated encephalopathy; BBB, Blood–brain barrier; NF-*κ*B, Nuclear factor kappa-light-chain-enhancer of activated B cells; NLRP3, NOD-, LRR- and pyrin domain-containing protein 3; LPS, Lipopolysaccharide; BMECs, Brain microvascular endothelial cells; ChIP, Chromatin immunoprecipitation; EMSA, Electrophoretic mobility shift assay; RNAP III, RNA polymerase (pol) III PTEN, Phosphatase and tensin homolog; CK-8, Cell Counting Kit-8; WT, wild-type; MUT, mutated; MWM, Morris water maze; ELISA, Enzyme-linked immunosorbent assay; SDS-PAGE, Sodium dodecyl sulfate-polyacrylamide gel electrophoresis; PVDF, Polyvinylidene fluoride.

## References

[1] Dario-Lucas Helbing LB Otto W Witte. Sepsis-associated encephalopathy. CMAJ (2018) 190(36):E1083. 10.1503/cmaj.180454 30201616 PMC6131085

[2] Ren C Yao R-q Zhang H Feng Y-w Yao Y-m Sepsis-associated encephalopathy: a vicious cycle of immunosuppression. J Neuroinflamm (2020) 17:14. 10.1186/s12974-020-1701-3 PMC6953314 31924221

[3] Gofton TE YG Sepsis-associated encephalopathy. Nat Rev Neurol (2012) 8:557–66. 10.1038/nrneurol.2012.183 22986430

[4] Gu M Mei XL Zhao YN Sepsis and Cerebral Dysfunction: BBB Damage, Neuroinflammation, Oxidative Stress, Apoptosis and Autophagy as Key Mediators and the Potential Therapeutic Approaches. Neurotox Res (2020). 10.1007/s12640-020-00270-5 32876918

[5] Sweeney MD Zhao Z Montagne A Nelson AR Zlokovic BV Blood-Brain Barrier: From Physiology to Disease and Back. Physiol Rev (2019) 99(1):21–78. 10.1152/physrev.00050.2017 30280653 PMC6335099

[6] Abbott NJ Patabendige AA Dolman DE Yusof SR Begley DJ Structure and function of the blood-brain barrier. Neurobiol Dis (2010) 37(1):13–25. 10.1016/j.nbd.2009.07.030 19664713

[7] Rhea EM Banks WA Role of the Blood-Brain Barrier in Central Nervous System Insulin Resistance. Front Neurosci-Switz (2019) 13:521. 10.3389/fnins.2019.00521 PMC6558081 31213970

[8] Jackson S Meeks C Vézina A Robey RW Tanner K Gottesman MM Model systems for studying the blood-brain barrier: Applications and challenges. Biomaterials (2019) 214:119217. 10.1016/j.biomaterials.2019.05.028 31146177

[9] Keep RF Zhou N Xiang J Andjelkovic AV Hua Y Xi G Vascular disruption and blood-brain barrier dysfunction in intracerebral hemorrhage. Fluids Barriers CNS (2014) 11:18. 10.1186/2045-8118-11-18. 10;11:18. 25120903 PMC4130123

[10] Sulhan S Lyon KA Shapiro LA Huang JH Neuroinflammation and blood-brain barrier disruption following traumatic brain injury: Pathophysiology and potential therapeutic targets. J Neurosci Res (2020) 98(1):19–28. 10.1002/jnr.24331 30259550 PMC6437022

[11] da Fonseca AC Matias D Garcia C Amaral R Geraldo LH Freitas C The impact of microglial activation on blood-brain barrier in brain diseases. Front Cell Neurosci (2014) 3:362(8):362. 10.3389/fncel.2014.00362 PMC4217497 25404894

[12] Lezoualc’h F Behl C Transcription factor NF-kappaB: friend or foe of neurons? Mol Psychiatry (1998) 3(1):15–20. 10.1038/sj.mp.4000295 9491808

[13] Islam MT Bardaweel SK Mubarak MS Koch W Gaweł-Beben W Antosiewicz B Immunomodulatory Effects of Diterpenes and Their Derivatives Through NLRP3 Inflammasome Pathway: A Review. Front Immunol (2020) 11:572136. 10.3389/fimmu.2020.572136 33101293 PMC7546345

[14] Chen C Wei YZ He XM Li DD Wang GQ Li JJ Naringenin Produces Neuroprotection Against LPS-Induced Dopamine Neurotoxicity via the Inhibition of Microglial NLRP3 Inflammasome Activation. Front Immunol (2019) 10:936. 10.3389/fimmu.2019.00936 31118933 PMC6504827

[15] Bu J Shi S Wang HQ Niu XS Zhao ZF Wu WD Acacetin protects against cerebral ischemia-reperfusion injury via the NLRP3 signaling pathway. Neural Regener Res (2019) 14(4):605–12. 10.4103/1673-5374.247465 PMC6352603 30632500

[16] Cao G Jiang N Hu Y Zhang YY Wang GY Yin MZ Ruscogenin Attenuates Cerebral Ischemia-Induced Blood-Brain Barrier Dysfunction by Suppressing TXNIP/NLRP3 Inflammasome Activation and the MAPK Pathway. Int J Mol Sci (2016) 17(9):1418. 10.3390/ijms17091418 PMC5037697 27589720

[17] Muñoz E-M Microglia-precursor cell interactions in health and in pathology. BIOCELL (2018) 42(2):41–6. 10.32604/biocell.2018.07011

[18] McAllister MJ Chemaly M Eakin AJ Gibson DS McGilligan VE NLRP3 as a potentially novel biomarker for the management of osteoarthritis. Osteoarthritis Cartilage (2018) 26(5):612–9. 10.1016/j.joca.2018.02.901 29499288

[19] Zhang C Boini KM Xia M Abais JM Li X Liu QL Activation of Nod-like receptor protein 3 inflammasomes turns on podocyte injury and glomerular sclerosis in hyperhomocysteinemia. Hypertension (Dallas Tex 1979) (2012) 60(1):154–62. 10.1161/HYPERTENSIONAHA.111.189688 PMC3753400 22647887

[20] Mridha AR Wree A Robertson AAB Yeh MM Johnon CD Van Ronnyen DM NLRP3 inflammasome blockade reduces liver inflammation and fibrosis in experimental NASH in mice. J Hepatol (2017) 66(5):1037–46. 10.1016/j.jhep.2017.01.022 PMC6536116 28167322

[21] Upadhya R Lee JH Willis IM Maf1 Is an Essential Mediator of Diverse Signals that Repress RNA Polymerase III Transcription. Mol Cell (2002) 10(6):1489–94. 10.1016/s1097-2765(02)00787-6 12504022

[22] Palian BM Rohira AD Johnson SA He L Zheng N Dubeau L Maf1 is a novel target of PTEN and PI3K signaling that negatively regulates oncogenesis and lipid metabolism. PloS Genet (2014) 11(3):e1005055. 10.1371/journal.pgen.1004789 PMC4263377 25502566

[23] Oficjalska-Pham D Harismendy O Smagowicz WJ Peredo AG Boguta M Sentenac A General Repression of RNA Polymerase III Transcription Is Triggered by Protein Phosphatase Type 2A-Mediated Dephosphorylation of Maf1. Mol Cell (2006) 22(5):623–32. 10.1016/j.molcel.2006.04.008 16762835

[24] Takata F Dohgu S Yamauchi A Matsumoto J Machida T Fujishta K In vitro blood-brain barrier models using brain capillary endothelial cells isolated from neonatal and adult rats retain age-related barrier properties. PloS One (2013) 8(1):e55166. 10.1371/journal.pone.0055166 23383092 PMC3561369

[25] Price GM Tien J Methods for forming human microvascular tubes in vitro and measuring their macromolecular permeability. Methods Mol Biol (Clifton NJ) (2011) 671:281–93. 10.1007/978-1-59745-551-0_17 20967637

[26] Radu M Chernoff J An in vivo assay to test blood vessel permeability. J Vis Exp (2013) 73:e50062. 10.3791/50062 PMC3639515 23524912

[27] Vorhees CV Williams MT Morris water maze: procedures for assessing spatial and related forms of learning and memory. Nat Protoc (2006) 1(2):848–58. 10.1038/nprot.2006.116 PMC2895266 17406317

[28] Banks WA Gray AM Erickson MA Salameh TS Damodarasamy M Sheibani N Lipopolysaccharide-induced blood-brain barrier disruption: roles of cyclooxygenase, oxidative stress, neuroinflammation, and elements of the neurovascular unit. J Neuroinflamm (2015) 12:223. 10.1186/s12974-015-0434-1 PMC4660627 26608623

[29] Kafa IM Uysal M Bakirci S Ayberk Kurt M Sepsis induces apoptotic cell death in different regions of the brain in a rat model of sepsis. Acta Neurobiol Exp (Wars) (2010) 70(3):246–60. 10.1021/cn900017w 20871644

[30] Danielski LG Giustina AD Bonfante S Barichello T Petronilho F The NLRP3 Inflammasome and Its Role in Sepsis Development. Inflammation (2020) 43(1):24–31. 10.1007/s10753-019-01124-9 31741197

[31] Wu J Zhang M Hao S Jia M Ji M Qiu LL Mitochondria-Targeted Peptide Reverses Mitochondrial Dysfunction and Cognitive Deficits in Sepsis-Associated Encephalopathy. Mol Neurobiol (2015) 52(1):783–91. 10.1007/s12035-014-8918-z 25288156

[32] Qiao Y Wang P Qi J Zhang L Gao C TLR-induced NF-kappaB activation regulates NLRP3 expression in murine macrophages. FEBS Lett (2012) 586(7):1022–6. 10.1016/j.febslet.2012.02.045 22569257

[33] Zhang S Li X Wang HY Steven Zheng XF Beyond regulation of pol III: Role of MAF1 in growth, metabolism, aging and cancer. Biochim Biophys Acta Gene Regul Mech (2018) 1861(4):338–43. 10.1016/j.bbagrm.2018.01.019 29407795

[34] Smith KR Oliver PL Lumb MJ Arancibia-Carcamo IL Revilla-Sanchez R Brandon NJ Identification and characterisation of a Maf1/Macoco protein complex that interacts with GABAA receptors in neurons. Mol Cell Neurosci (2010) 44(4):330–41. 10.1016/j.mcn.2010.04.004 PMC2931578 20417281

[35] Sun Y Chen C Xue R Wang Y Dong B Li JY Maf1 ameliorates cardiac hypertrophy by inhibiting RNA polymerase III through ERK1/2. Theranostics (2019) 9(24):7268–81. 10.7150/thno.33006 PMC6831308 31695767

[36] Kuperberg SJ Wadgaonkar R Sepsis-Associated Encephalopathy: The Blood-Brain Barrier and the Sphingolipid Rheostat. Front Immunol (2017) 8:597. 10.3389/fimmu.2017.00597 28670310 PMC5472697

[37] Chow BW Gu C The molecular constituents of the blood-brain barrier. Trends Neurosci (2015) 38(10):598–608. 10.1016/j.tins.2015.08.003 26442694 PMC4597316

[38] Sandoval KE Witt KA Blood-brain barrier tight junction permeability and ischemic stroke. Neurobiol Dis (2008) 32(2):200–19. 10.1016/j.nbd.2008.08.005 18790057

[39] Yan J Li L Khatibi NH Yang L Wang K Zhang WG Blood-brain barrier disruption following subarchnoid hemorrhage may be faciliated through PUMA induction of endothelial cell apoptosis from the endoplasmic reticulum. Exp Neurol (2011) 230(2):240–7. 10.1016/j.expneurol.2011.04.022 21586287

[40] Yende S D’Angelo G Kellum JA Weissfeld L Fine J Welch RD Inflammatory markers at hospital discharge predict subsequent mortality after pneumonia and sepsis. Am J Respir Crit Care Med (2008) 177(11):1242–7. 10.1164/rccm.200712-1777OC PMC2720087 18369199

[41] Erickson MA Banks WA Cytokine and chemokine responses in serum and brain after single and repeated injections of lipopolysaccharide: multiplex quantification with path analysis. Brain Behav Immun (2011) 25(8):1637–48. 10.1016/j.bbi.2011.06.006 PMC3389494 21704698

[42] Annane D Sharshar T Cognitive decline after sepsis. Lancet Respir Med (2015) 3(1):61–9. 10.1016/S2213-2600(14)70246-2 25434614

[43] Hise AG Tomalka J Ganesan S Patel K Hall BA Brown GD An essential role for the NLRP3 inflammasome in host defense against the human fungal pathogen Candida albicans. Cell Host Microbe (2009) 5(5):487–97. 10.1016/j.chom.2009.05.002 PMC2824856 19454352

[44] Fernandes-Alnemri T Wu J Yu JW Datta P Miller B Jankowski W The pyroptosome: a supramolecular assembly of ASC dimers mediating inflammatory cell death via caspase-1 activation. Cell Death Differ (2007) 14(9):1590–604. 10.1038/sj.cdd.4402194 PMC3345951 17599095

[45] Hoss F Rodriguez-Alcazar JF Latz E Assembly and regulation of ASC specks. Cell Mol Life Sci CMLS (2017) 74(7):1211–29. 10.1007/s00018-016-2396-6 PMC11107573 27761594

[46] Kohman RA Rhodes JS Neurogenesis, inflammation and behavior. Brain Behav Immun (2013) 27(1):22–32. 10.1016/j.bbi.2012.09.003 22985767 PMC3518576

[47] Kim EJ Park SY Baek SE Jang MA Lee WS Bae SS HMGB1 Increases IL-1β Production in Vascular Smooth Muscle Cells via NLRP3 Inflammasome. Front Physiol (2018) 9:313:313. 10.3389/fphys.2018.00313 29643819 PMC5882820

[48] Yang F Wang Z Wei X Han H Meng X Zhang Y NLRP3 deficiency ameliorates neurovascular damage in experimental ischemic stroke. J Cereb Blood Flow Metab (2014) 34(4):660–7. 10.1038/jcbfm.2013.242 PMC3982086 24424382

[49] Wang H Chen H Jin J Liu Q Zhong D Li G Inhibition of the NLRP3 inflammasome reduces brain edema and regulates the distribution of aquaporin-4 after cerebral ischaemia-reperfusion. Life Sci (2020) 251:117638. 10.1016/j.lfs.2020.117638 32251636

[50] Qu XY Zhang YM Tao LN Gao H Zhai JH Sun JM XingNaoJing injections protect against cerebral ischemia/reperfusion injury and alleviate blood-brain barrier disruption in rats, through an underlying mechanism of NLRP3 inflammasomes suppression. Chin J Nat Med (2019) 17(7):498–505. 10.1016/S1875-5364(19)30071-8 31514981

[51] Xu F Shen G Su Z He Z Yuan L Glibenclamide ameliorates the disrupted blood-brain barrier in experimental intracerebral hemorrhage by inhibiting the activation of NLRP3 inflammasome. Brain Behav (2019) 9(4):e01254. 10.1002/brb3.1254 30859754 PMC6456786

[52] Dong Y Fan C Hu W Jiang S Ma ZQ Yan XL Melatonin attenuated early brain injury induced by subarachnoid hemorrhage via regulating NLRP3 inflammasome and apoptosis signaling. J Pineal Res (2016) 60(3):253–62. 10.1111/jpi.12300 26639408

[53] Zhong X Xie L Yang X Liang F Yang Y Tong J Ethyl pyruvate protects against sepsis-associated encephalopathy through inhibiting the NLRP3 inflammasome. Mol Med (2020) 26(1):55. 10.1186/s10020-020-00181-3 32517686 PMC7285451

[54] Tsuchiya K Inflammasome-associated cell death: Pyroptosis, apoptosis, and physiological implications. Microbiol Immunol (2020) 64(4):252–69. 10.1111/1348-0421.12771 31912554

[55] Shi CS Kehrl JH Cytochrome c Negatively Regulates NLRP3 Inflammasomes. PloS One (2016) 11(12):e0167636. 10.1371/journal.pone.0167636 28030552 PMC5193325

[56] Zhou R Yazdi AS Menu P Tschopp J A role for mitochondria in NLRP3 inflammasome activation. Nature (2011) 469(7329):221–5. 10.1038/nature09663 21124315

[57] Stutz A Kolbe CC Stahl R Horvath G Franklin BS Ray OV NLRP3 inflammasome assembly is regulated by phosphorylation of the pyrin domain. J Exp Med (2017) 214(6):1725–36. 10.1084/jem.20160933 PMC5460996 28465465

[58] Kelley N Jeltema D Duan Y He Y The NLRP3 Inflammasome: An Overview of Mechanisms of Activation and Regulation. Int J Mol Sci (2019) 20(13):3328. 10.3390/ijms20133328 PMC6651423 31284572

[59] Chen X Wang N Zhu Y Lu Y Liu X Zheng J The Antimalarial Chloroquine Suppresses LPS-Induced NLRP3 Inflammasome Activation and Confers Protection against Murine Endotoxic Shock. Mediators Inflamm (2017) 2017:6543237. 10.1155/2017/6543237 28321151 PMC5340938

[60] Opal SM Endotoxins and other sepsis triggers. Contrib Nephrol (2010) 167:14–24. 10.1159/000315915 20519895

[61] Lin CC Hsieh HL Shih RH Chi PL Cheng SE Yang CM Up-regulation of COX-2/PGE2 by endothelin-1 via MAPK-dependent NF-kappaB pathway in mouse brain microvascular endothelial cells. Cell Commun Signal CCS (2013) 11(1):8. 10.1186/1478-811X-11-8 23343326 PMC3560266

[62] Boaru SG Borkham-Kamphorst E Van de Leur E Lehnen E Liedtke C Weiskirchen R NLRP3 inflammasome expression is driven by NF-kappaB in cultured hepatocytes. Biochem Biophys Res Commun (2015) 458(3):700–6. 10.1016/j.bbrc.2015.02.029 25686493

[63] Liang X Zhang D Liu W Yan Y Zhou F Wu W Reactive oxygen species trigger NF-kappaB-mediated NLRP3 inflammasome activation induced by zinc oxide nanoparticles in A549 cells. Toxicol Ind Health (2017) 33(10):737–45. 10.1177/0748233717712409 28870124

[64] Fann DY Lim YA Cheng YL Lok KZ Chunduri P Baik SH Evidence that NF-kappaB and MAPK Signaling Promotes NLRP Inflammasome Activation in Neurons Following Ischemic Stroke. Mol Neurobiol (2018) 55(2):1082–96. 10.1007/s12035-017-0394-9 28092085

[65] Vannini A Ringel R Kusser AG Berninghausen O Kassavetis GA Cramer P Molecular basis of RNA polymerase III transcription repression by Maf1. Cell (2010) 143(1):59–70. 10.1016/j.cell.2010.09.002 20887893

[66] Rollins J Veras I Cabarcas S Willis I Schramm L Human Maf1 negatively regulates RNA polymerase III transcription via the TFIIB family members Brf1 and Brf2. Int J Biol Sci (2007) 3(5):292–302. 10.7150/ijbs.3.292 17505538 PMC1865091

[67] Orioli A Praz V Lhote P Hernandez N Human MAF1 targets and represses active RNA polymerase III genes by preventing recruitment rather than inducing long-term transcriptional arrest. Genome Res (2016) 26(5):624–35. 10.1101/gr.201400.115 PMC4864463 26941251

