# Supplementary figures and images for: Maf1 Ameliorates Sepsis-Associated Encephalopathy by Suppressing the NF-kB/NLRP3 Inflammasome Signaling Pathway
Source: Front Immunol. 2020 Dec 23;11:594071. doi: 10.3389/fimmu.2020.594071 (PMC7785707; doi:10.3389/fimmu.2020.594071)

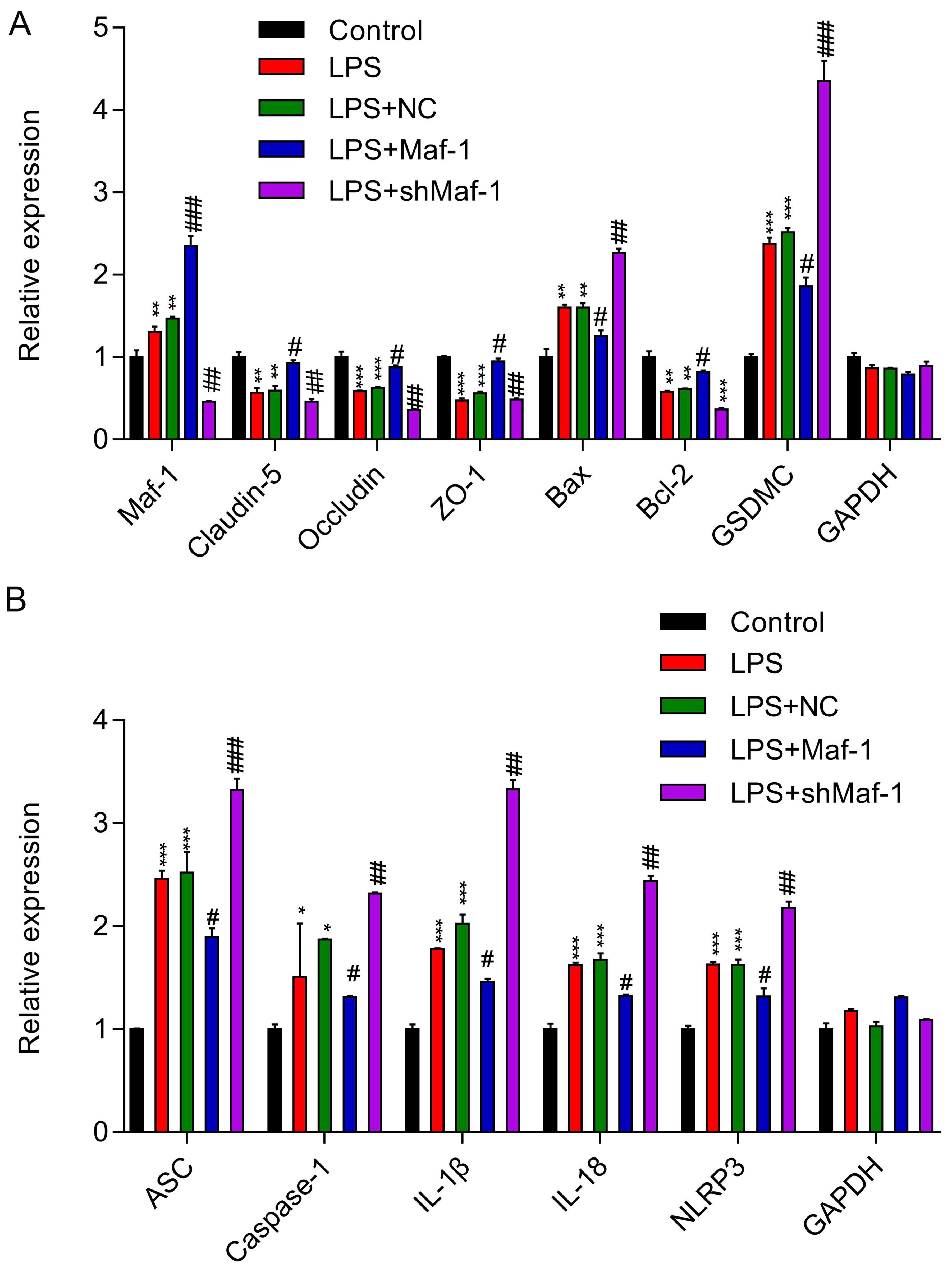

Supplement: Supplementary Figure 1, related to Figure 1 — (B, C). A summary of western blot results from three independent experiments. **, p < 0.01 and ***, p < 0.001, compared with Control; #, p < 0.05, ##, p < 0.01 and ###, p < 0.001, compared with LPS + NC. [file Image_1.jpeg]

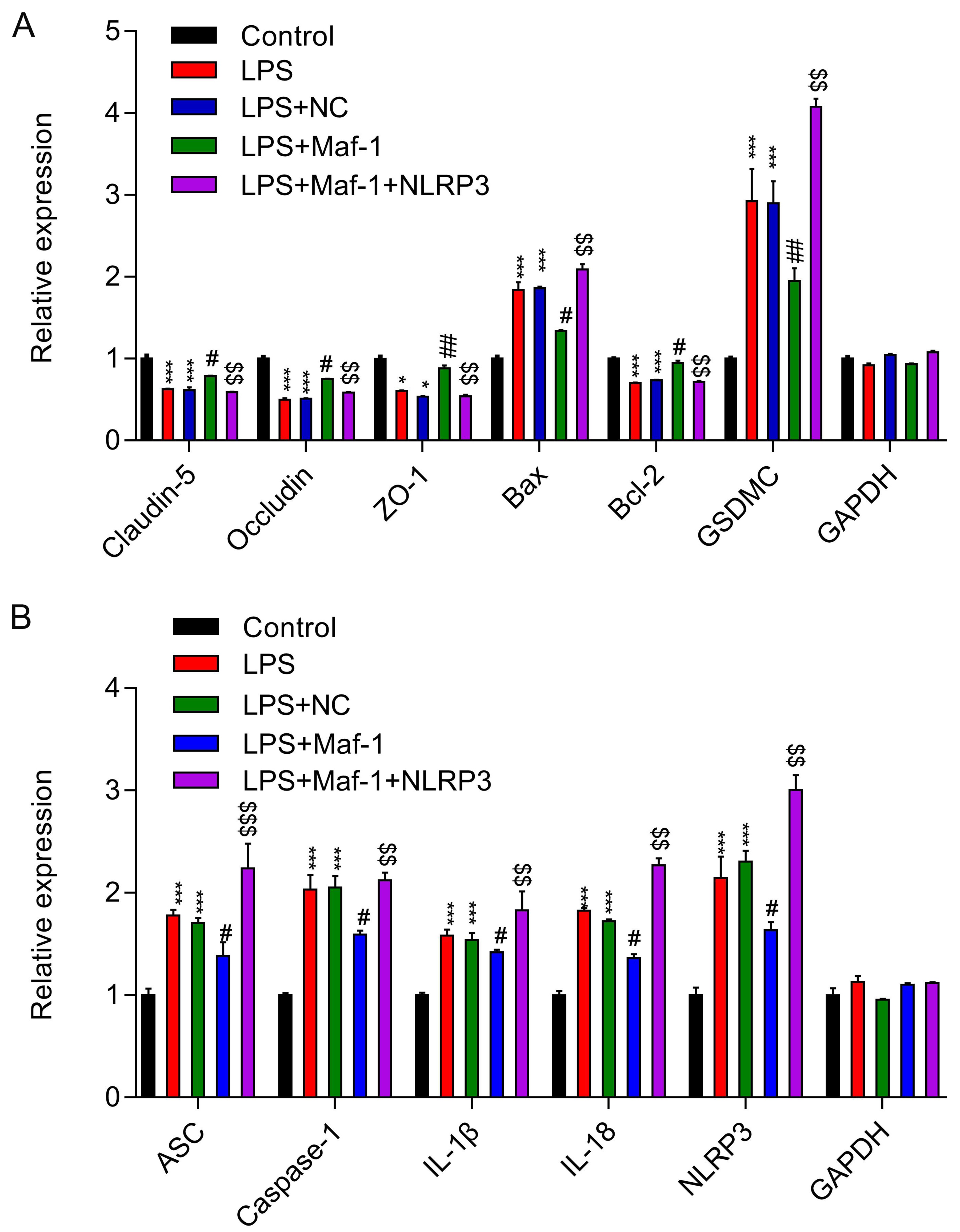

Supplement: Supplementary Figure 2, related to Figure 2 — (B, C). A summary of western blot results from three independent experiments. *, p < 0.01 and ***, p < 0.001, compared with Control; #, p<0.05 and ##, p<0.01, compared with LPS + NC; $$, p < 0.01 and $$$, p < 0.001, compared with LPS+Maf-1. [file Image_2.jpeg]

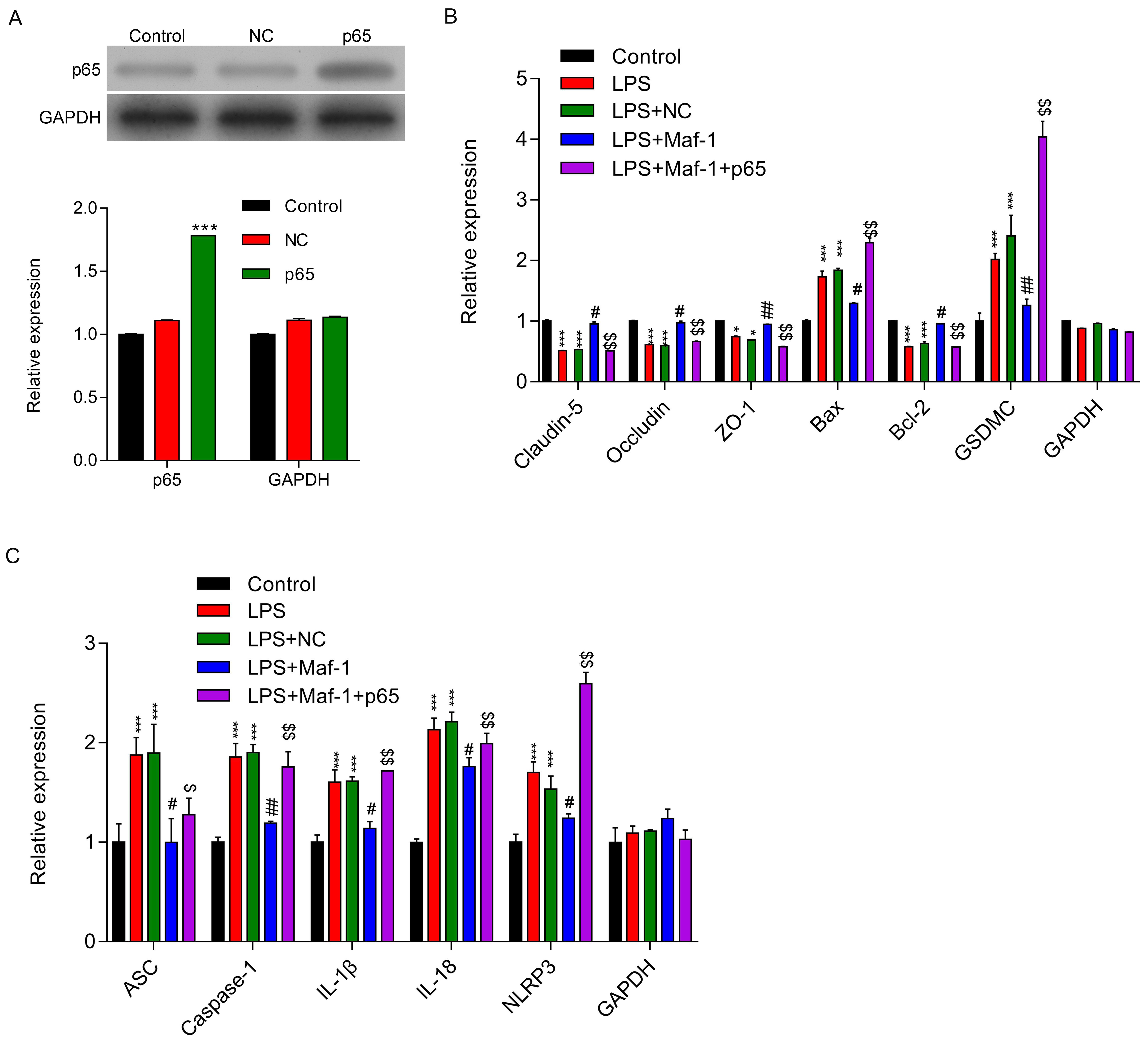

Supplement: Supplementary Figure 3, related to Figure 3 — (A). Expression of p65 after forcing overexpressionof p65 in BMSCs. (B, C). A summary of western blot results from three independent experiments. ***, p < 0.001, compared with Control; #, p < 0.05 and ##, p < 0.01, compared with LPS+NC; $, p < 0.05 and $$, p < 0.01, compared with LPS+Maf-1. [file Image_3.jpeg]

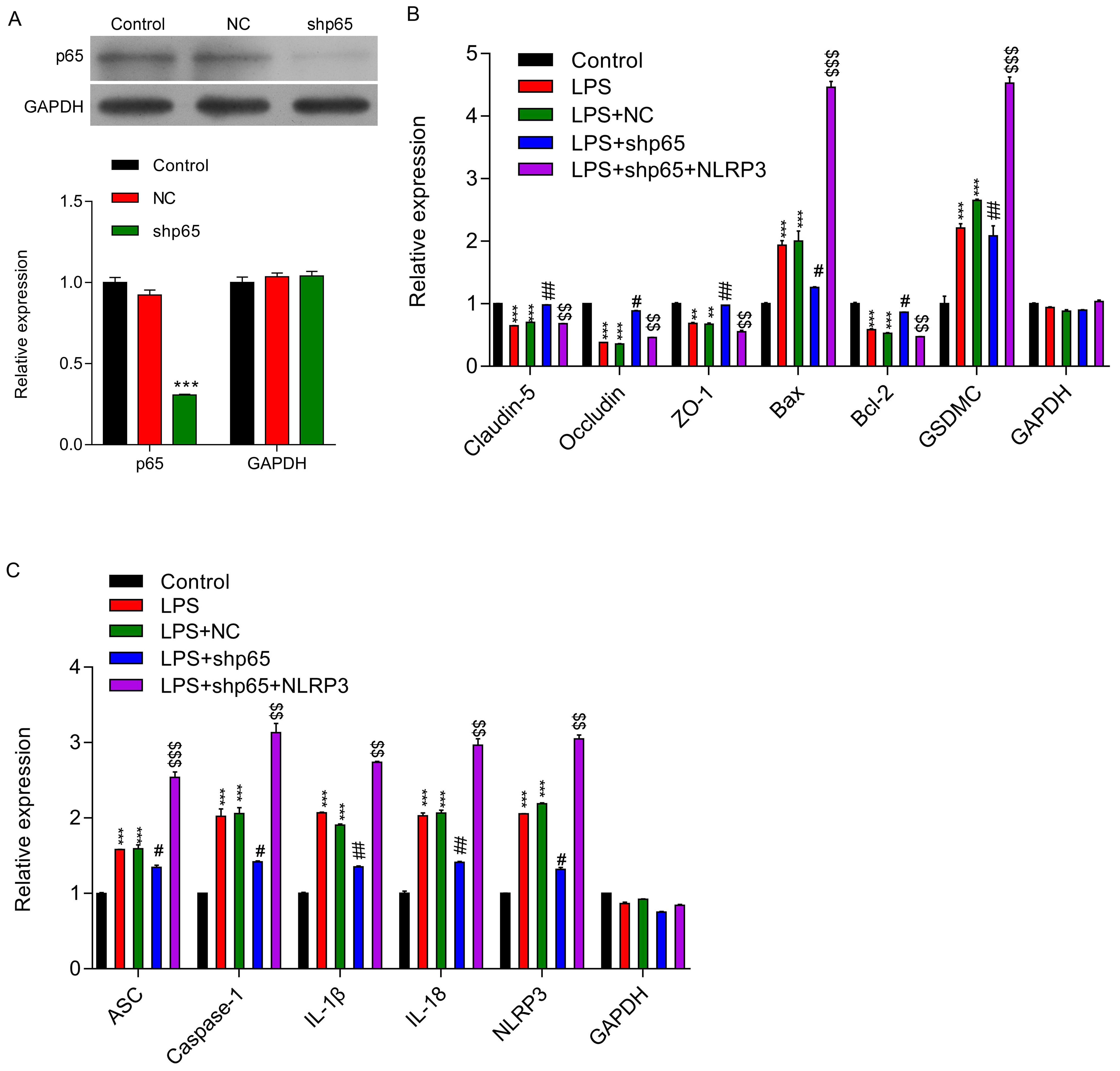

Supplement: Supplementary Figure 4, related to Figure 5 — (A). Expression of p65 after suppressing p65 expression in BMSCs. (B, C). A summary of western blot results from three independent experiments. **, p < 0.01 and ***, p < 0.001, compared with Control; #, p < 0.05 and ##, p < 0.01, compared with LPS + NC; $$, p < 0.01 and $$$, p < 0.001, compared with LPS + shp65. [file Image_4.jpeg]

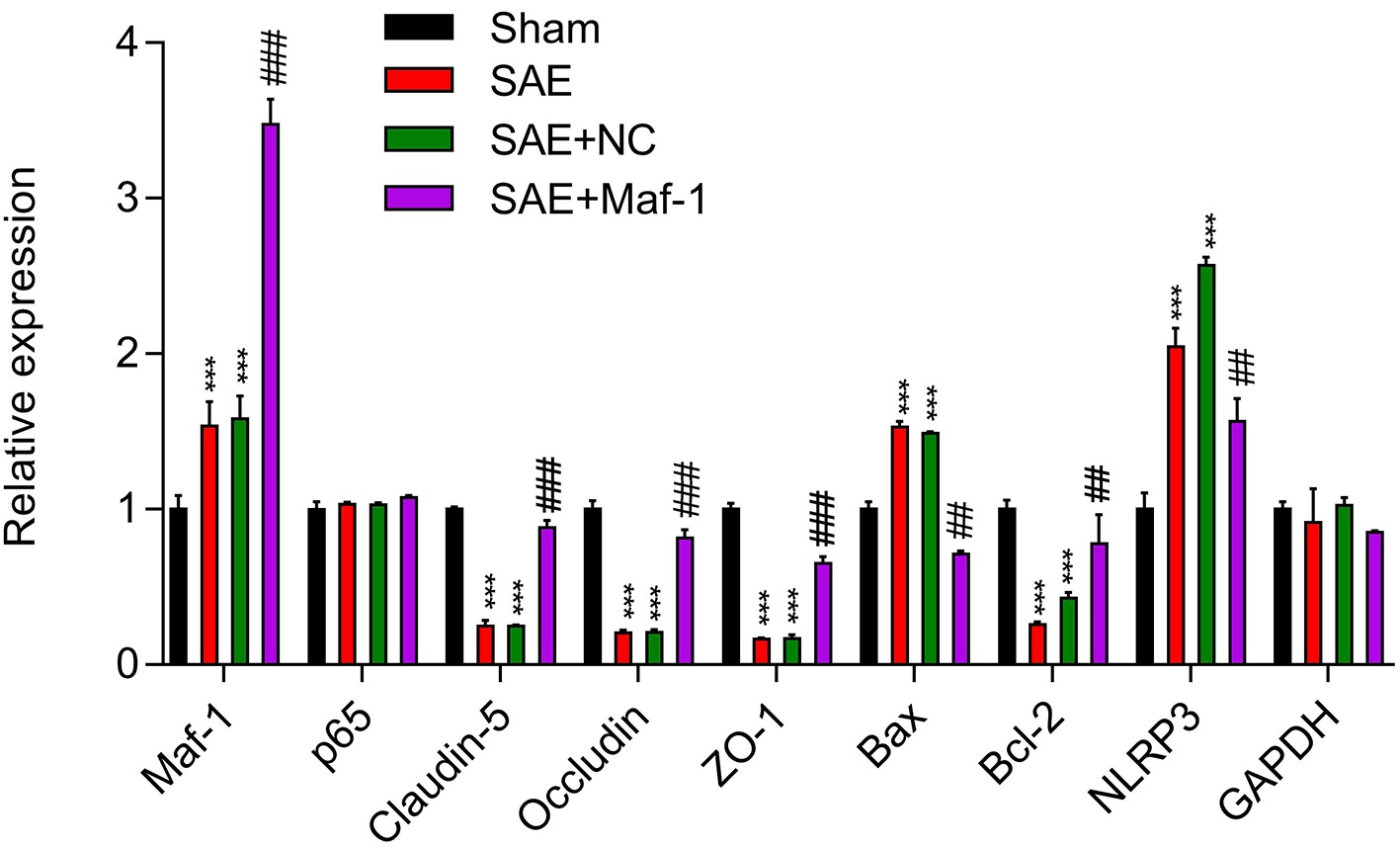

Supplement: Supplementary file 5 [file Image_5.jpeg]
