# Supplementary material for: Maf1 Ameliorates Sepsis-Associated Encephalopathy by Suppressing the NF-kB/NLRP3 Inflammasome Signaling Pathway
Source: Front Immunol. 2020 Dec 23;11:594071. doi: 10.3389/fimmu.2020.594071 (PMC7785707; doi:10.3389/fimmu.2020.594071)

Original images

Figure 1

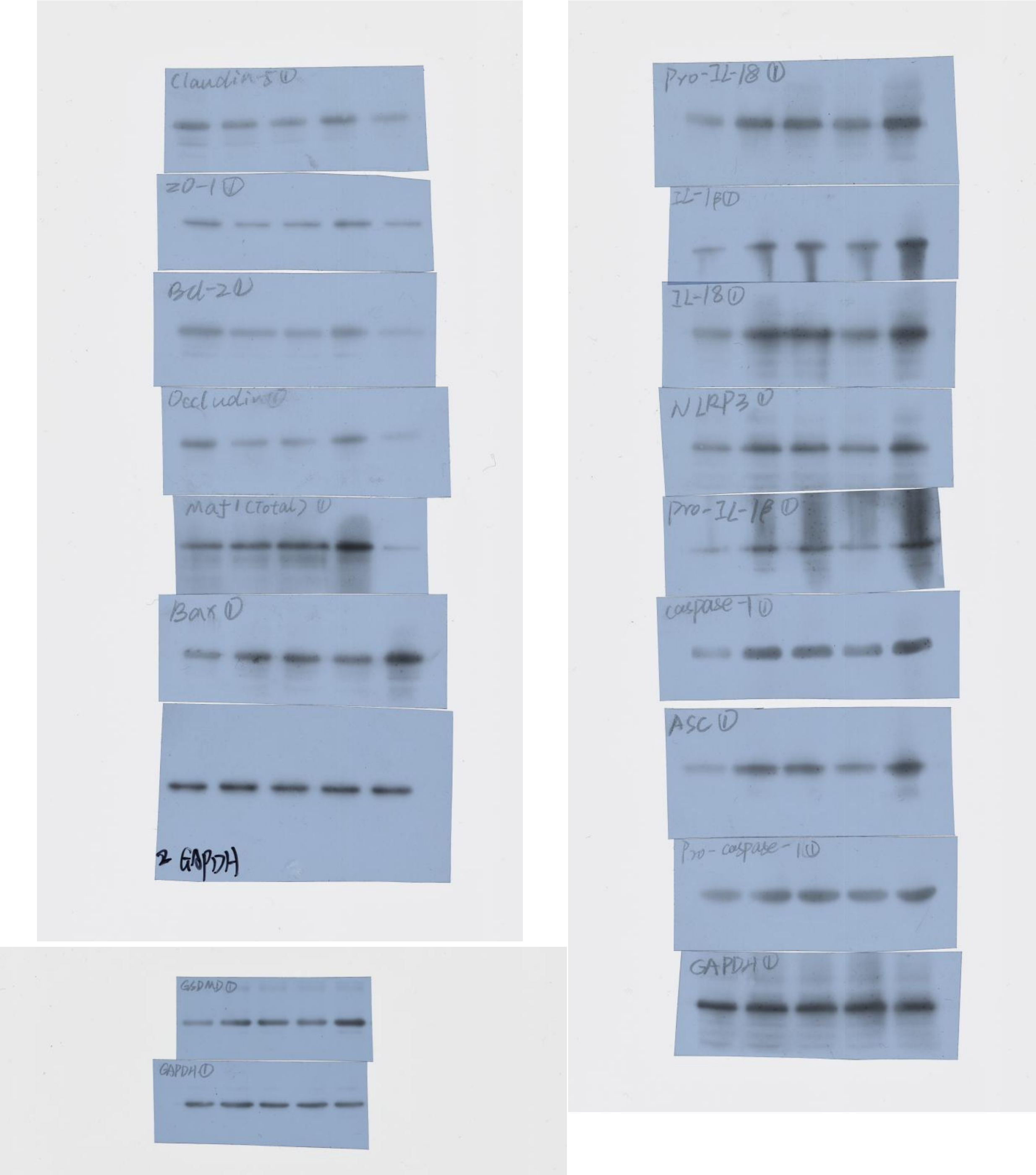

Figure 2

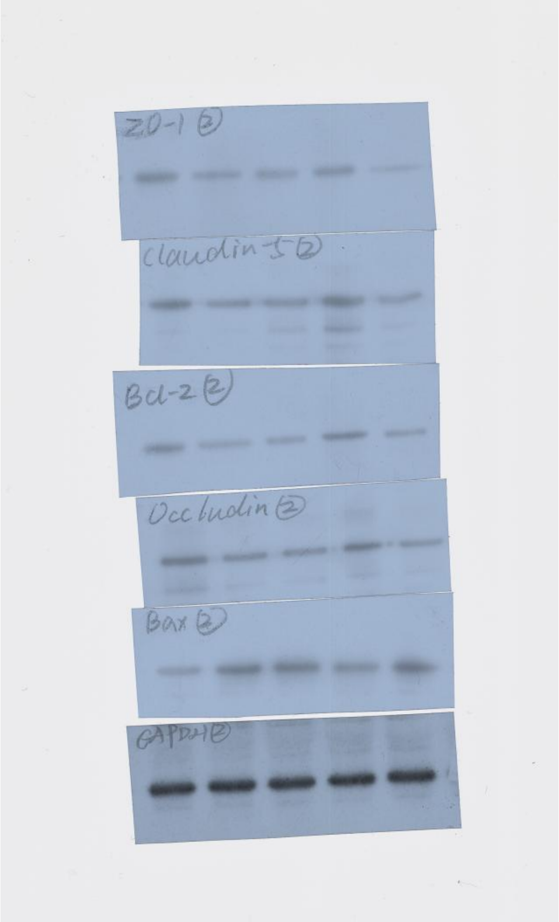
ZO-1
Claudin-5
Bcl-2
Occludin
Bax
GAPDH

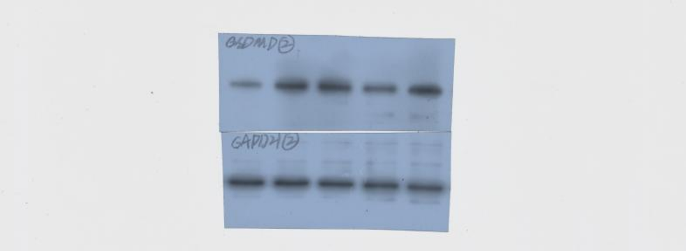
GSDMD
GAPDH

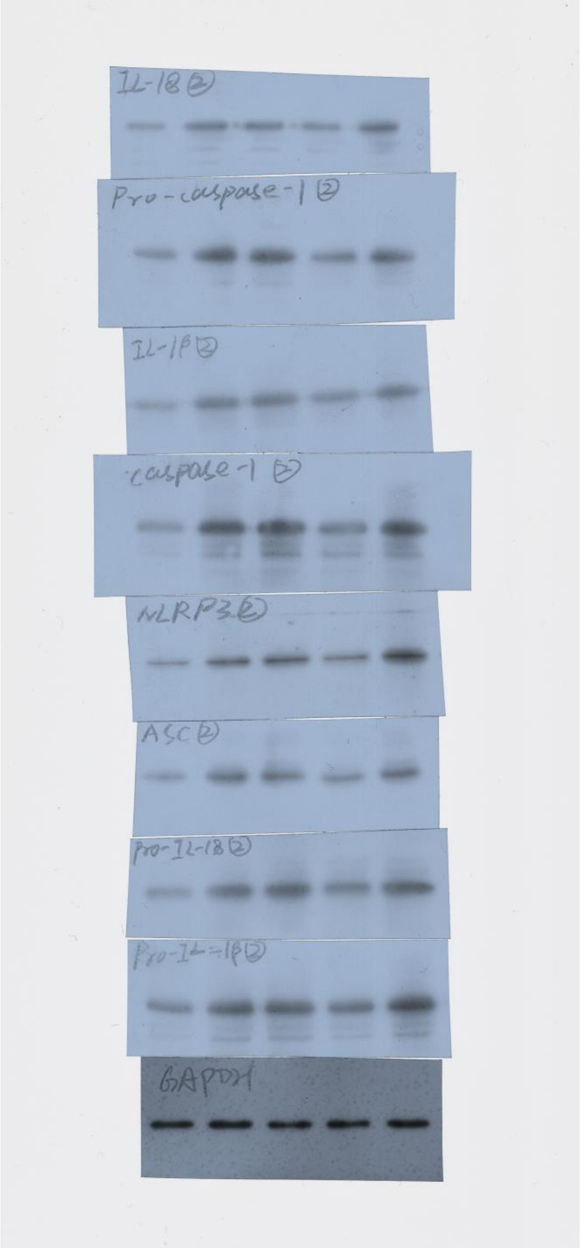
IL-18
Pro-caspase-1
IL-1β
caspase-1
NLRP3
ASC
Pro-IL-18
Pro-IL-1β
GAPDH

Figure 3

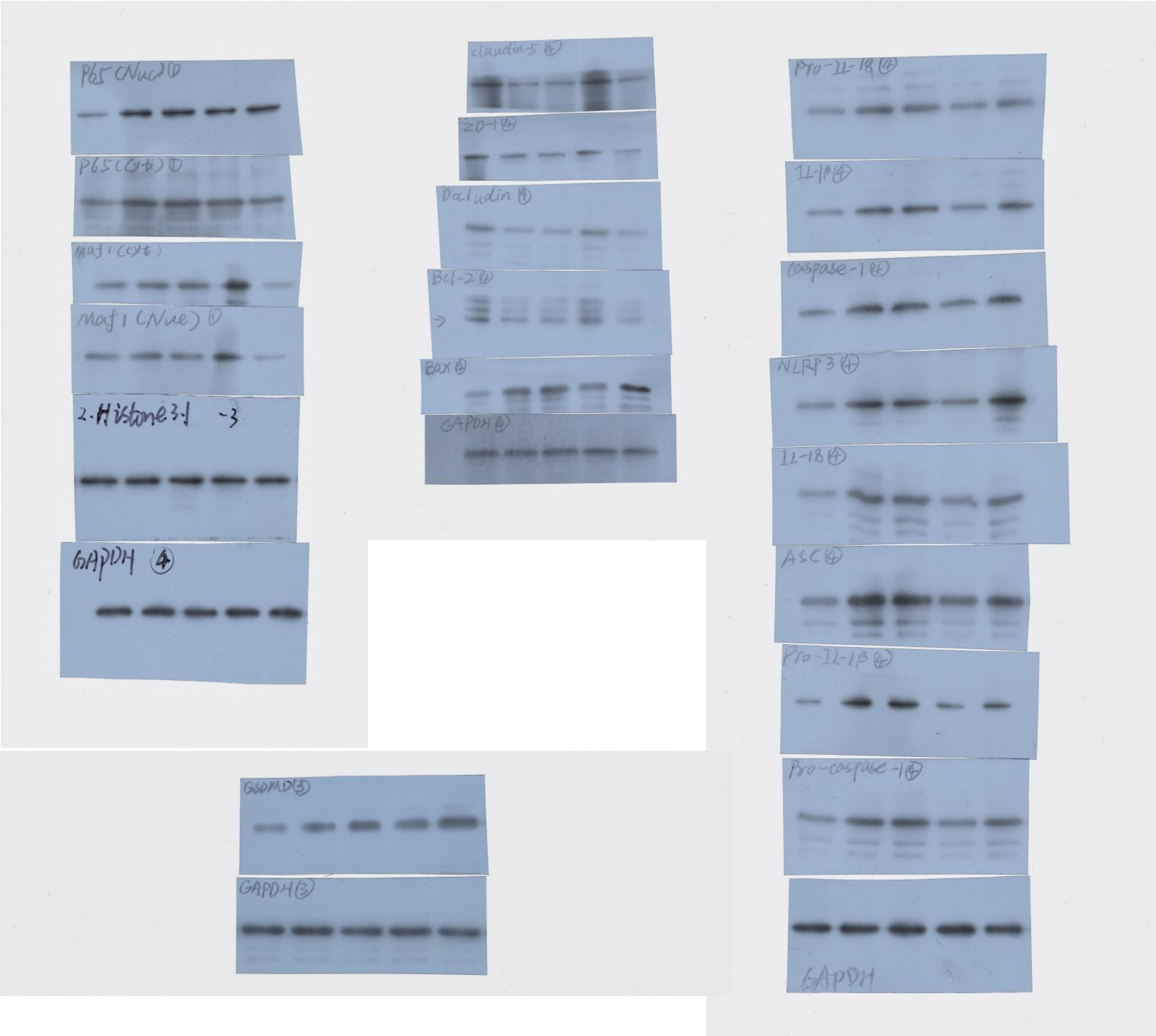
P65 (Nuc)
P65 (Cyt)
Maf1 (cyt)
Maf1 (Nuc)
Histone 3
GAPDH
claudin-5
ZO-1
Occludin
Bcl-2
Bax
GAPDH
GSDMD
GAPDH
Pro-IL-18
IL-1β
caspase-1
NLRP3
IL-18
ASC
Pro-IL-1β
Pro-caspase-1
GAPDH

Figure 4

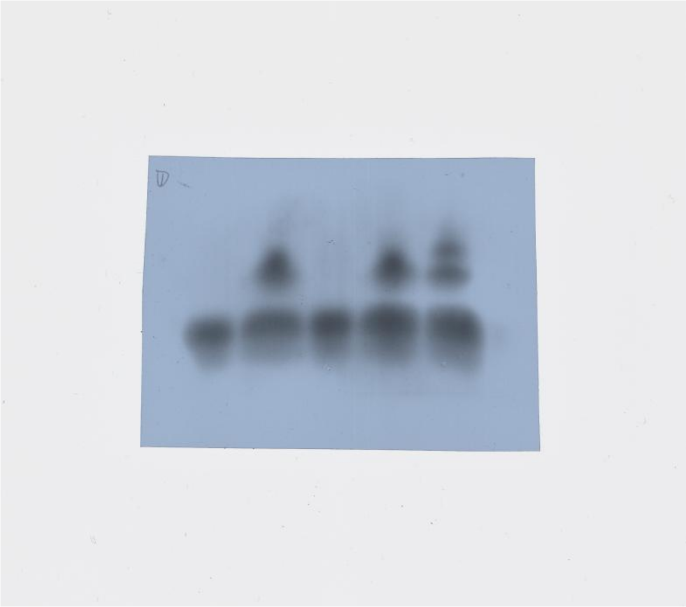

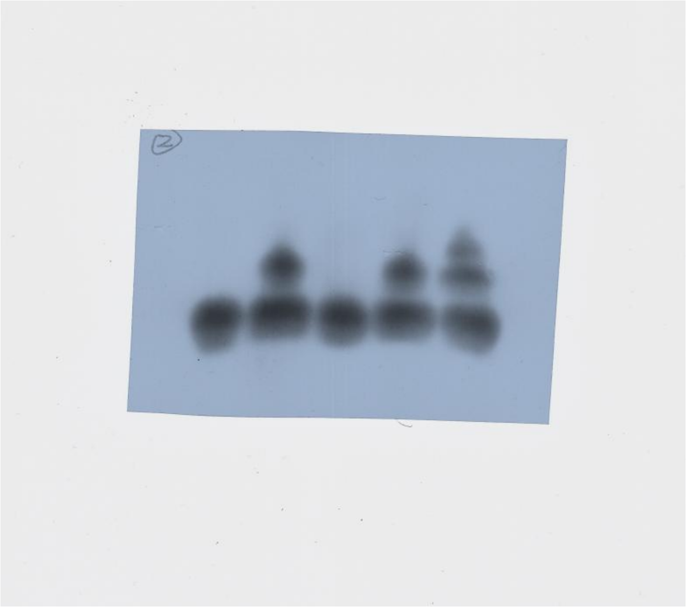

Figure 5

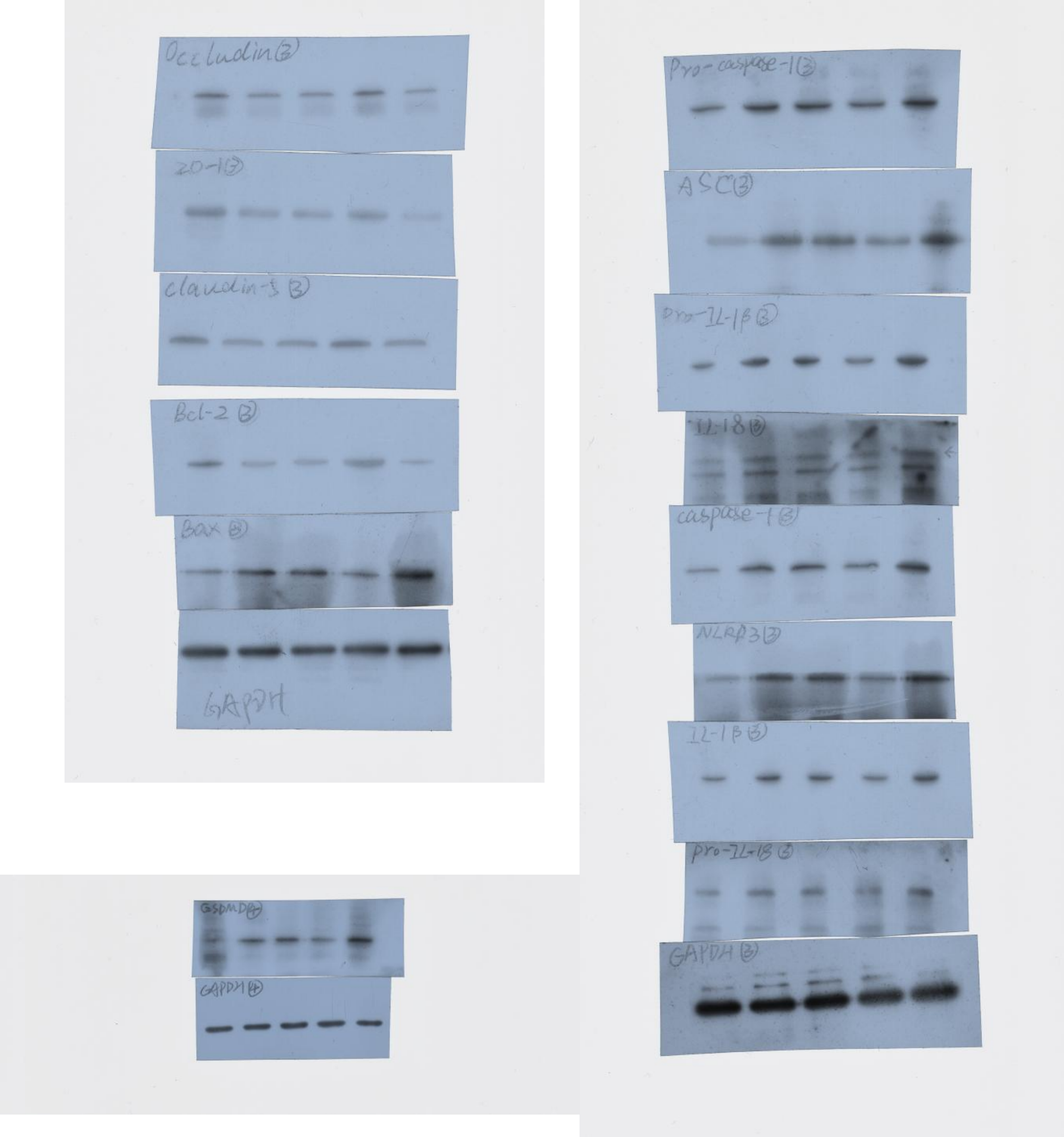
Occludin
ZO-1
claudin-3
Bcl-2
Bax
GAPDH
Pro-caspase-1
ASC
Pro-IL-1β
IL-18
caspase-1
NLRP3
IL-1β
GAPDH
GSDMD
GAPDH

Figure 6

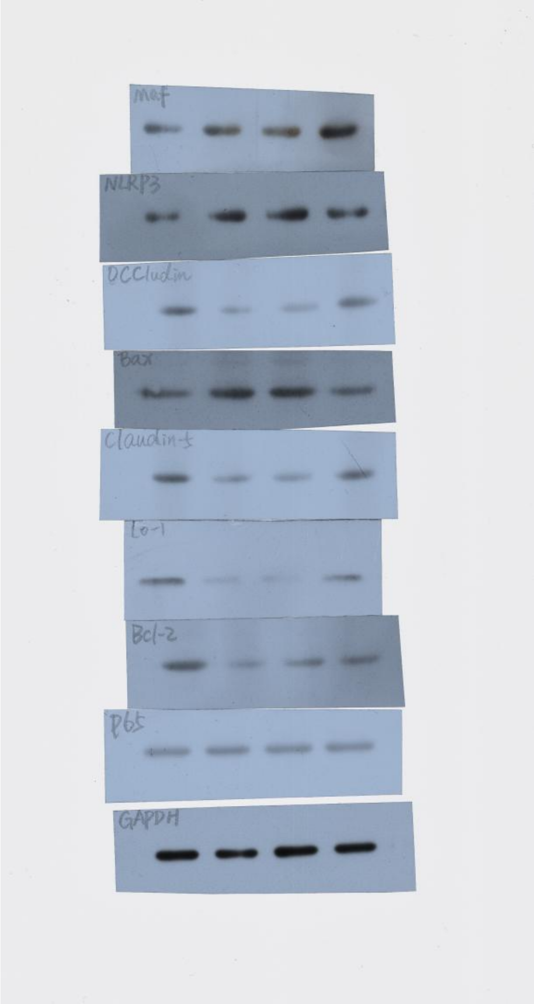

Supplementary Figure 3

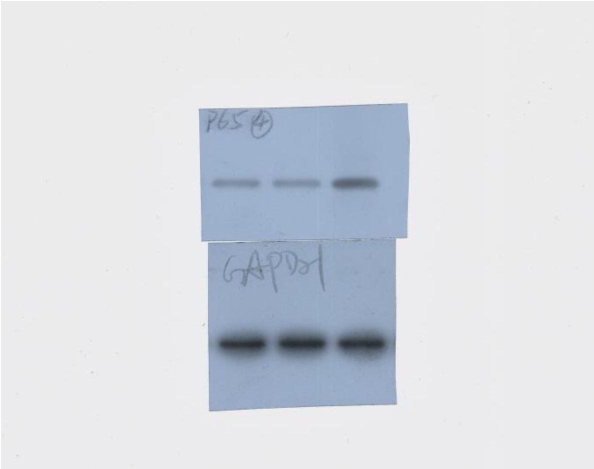

Supplementary Figure 4

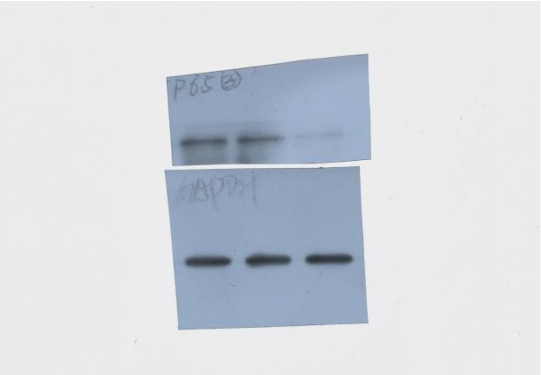

Supplement: Supplementary file 6 [file DataSheet_1.pdf]
